# Suspect screening and targeted analysis of acyl coenzyme A thioesters in bacterial cultures using a high-resolution tribrid mass spectrometer

**DOI:** 10.1007/s00216-021-03318-3

**Published:** 2021-04-21

**Authors:** Nevenka Cakić, Bernd Kopke, Ralf Rabus, Heinz Wilkes

**Affiliations:** 1 Organic Geochemistry, Carl von Ossietzky University Oldenburg, 26129 Oldenburg, Germany; 2 General & Molecular Microbiology, Institute for Chemistry and Biology of the Marine Environment (ICBM), Carl von Ossietzky University Oldenburg, 26129 Oldenburg, Germany

**Keywords:** Orbitrap Fusion, Acyl coenzyme A thioesters, Method development, Metabolic pathways, Betaproteobacterium “*Aromatoleum*” sp. strain HxN1

## Abstract

Analysis of acyl coenzyme A thioesters (acyl-CoAs) is crucial in the investigation of a wide range of biochemical reactions and paves the way to fully understand the concerned metabolic pathways and their superimposed networks. We developed two methods for suspect screening of acyl-CoAs in bacterial cultures using a high-resolution Orbitrap Fusion tribrid mass spectrometer. The methods rely on specific fragmentation patterns of the target compounds, which originate from the coenzyme A moiety. They make use of the formation of the adenosine 3′,5′-diphosphate key fragment (*m*/*z* 428.0365) and the neutral loss of the adenosine 3′-phosphate-5′-diphosphate moiety (506.9952) as preselection criteria for the detection of acyl-CoAs. These characteristic ions are generated either by an optimised in-source fragmentation in a full scan Orbitrap measurement or by optimised HCD fragmentation. Additionally, five different filters are included in the design of method. Finally, data-dependent MS/MS experiments on specifically preselected precursor ions are performed. The utility of the methods is demonstrated by analysing cultures of the denitrifying betaproteobacterium “*Aromatoleum*” sp. strain HxN1 anaerobically grown with hexanoate. We detected 35 acyl-CoAs in total and identified 24 of them by comparison with reference standards, including all 9 acyl-CoA intermediates expected to occur in the degradation pathway of hexanoate. The identification of additional acyl-CoAs provides insight into further metabolic processes occurring in this bacterium. The sensitivity of the method described allows detecting acyl-CoAs present in biological samples in highly variable abundances.

**Supplementary Information:**

The online version contains supplementary material available at 10.1007/s00216-021-03318-3.

**Figure:**
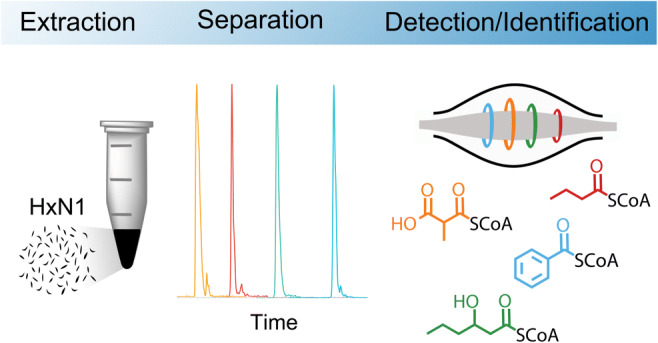
Graphical abstract

## Introduction

Acyl coenzyme A thioesters (acyl-CoAs) possess an energy-rich thioester bond, which can trigger carbon-carbon bond activation and its cleavage in a diverse range of enzymatic reactions. Due to these properties, acyl-CoAs are intermediates in many different metabolic processes such as β-oxidation of fatty acids, amino acid degradation and the tricarboxylic acid cycle. Acyl-CoAs are also important in secondary metabolism, like biosynthesis of polyketide and non-ribosomal peptide antibiotics [1, 2]. Profiling of acyl-CoAs thus serves as an important tool for the better understanding of metabolic pathways, including but not limited to environmentally and biomedically relevant processes [3–5]. Therefore, unambiguous identification of acyl-CoAs against the background of complex sample matrices is of high relevance and represents a great challenge in metabolomics research.

Among the techniques used for the detection and identification of acyl-CoAs, the one utilised most extensively is high-performance liquid chromatography (HPLC) coupled with mass spectrometry (MS). In contrast to more or less standardised MS methods, the HPLC methods applied in these approaches may be rather variable, especially with respect to the eluents used, which also may have a direct influence on the separation of isomers. The importance of isomer separation has already been discussed, highlighting the fact that one-third of naturally occurring CoA thioesters currently listed in the KEGG database are isobaric in terms of nominal masses [6]. Notably, such compounds are easily distinguishable by high-resolution mass spectrometry if they have different elemental compositions and, moreover, could be expected to show different retention behaviours as well. To date, the separation of isomeric acyl-CoAs, for example methylmalonyl- and succinyl-CoA as well as butyryl- and isobutyryl-CoA, was achieved by either using ion-pairing reagents [6, 7] or non-standard two-dimensional LC-MS [8]. The former is broadly used for the analysis of acyl-CoAs, especially in conjunction with electrospray ionisation in negative mode, since ion-pairing reagents can cause ion suppression in positive mode [6, 9]. The optimisation of HPLC methods, allowing isomer separation but not affecting MS, is still of high demand.

Identification of acyl-CoAs is mainly based on the detection of molecular ions, cleavage rules or even prediction of retention rules of some acyl-CoAs in a mixture [6, 8, 10]. The very specific fragmentation patterns of acyl-CoAs allow their selective analysis in different biological matrices [2, 7, 10]. Reversed-phase LC coupled to a triple quadrupole mass spectrometer has been widely used in targeted and non-targeted screening measurements. In both cases, the only selection applied was a fragment arising from a characteristic neutral loss of acyl-CoAs. For targeted experiments, it was the basis of multiple reaction monitoring (MRM) [2, 7, 10, 11], while a precursor ion scanning method based on this characteristic fragment (the neutral loss scan mode) has been applied in non-targeted experiments [6, 12, 13]. Although it has been shown that the adenosine 3′,5′-diphosphate key fragment (*m*/*z* 428.0365) is common to all thioesters [2], it has not been used for targeted screening on triple quadrupole instruments in MRM mode when only one transition was used [14]. Since other metabolites can also split off an adenosine diphosphate fragment (e.g. ATP, NAD, FAD) with *m*/*z* 428, this fragment is not exclusively representative for acyl-CoA thioesters and it was used for additional qualification only in QQQ targeted screenings [15]. However, it still functions as a tool to diagnose the presence of acyl-CoAs by generating a selected ion chromatogram of this ion [2].

High-resolution accurate mass (HRAM) spectrometers such as Q-TOF and Orbitrap have been also used for the analysis of acyl-CoAs [8, 9]. Lately, a lot of different methods for the identification of acyl-CoAs in complex biological matrices have been developed for the Orbitrap instruments [8, 16–19]. Typical operation mode of these instruments is acquisition of high-resolution full scan spectra in the Orbitrap and simultaneous conduction of data-dependent MS/MS experiments (ddMS^2^) in the ion trap, facilitating identification of unknowns. The method has been applied to the analysis of a range of different acyl-CoAs in biological samples, relying on detection of molecular ions and characteristic fragments [8]. In some cases, only high-resolution full scan measurement was used. In one study, the goal was to identify as many metabolites as possible, including but not limited to acyl-CoAs [16], while in the other, it was important to detect the acyl-CoAs expected to occur during microbial assimilation of methane [17]. Targeted MS/MS profiling of acyl-CoAs on an Orbitrap hybrid instrument has also been reported, using the presence of typical fragments as an important principle for their identification. Interestingly, in the mass spectra of almost all detected acyl-CoAs, the characteristic fragments *m*/*z* 136 and *m*/*z* 261 were reported, but *m*/*z* 428 in a very few only [18]. This may be due to the application of different collision energies for targeted MS^2^ scans and classic suspected screenings at which the *m*/*z* 428 fragment (as the precursor of the *m*/*z* 136 fragment) may occur only insignificantly. In the majority of the abovementioned studies, Q-Exactive Orbitrap mass spectrometers were used [8, 16, 18]. Recently, the first Orbitrap tribrid mass spectrometer instrument, Orbitrap Fusion, was introduced, which is capable of achieving mass resolutions of 500,000 (at *m*/*z* 200, FWHM). Since then, this high-resolution instrument has been increasingly applied for proteomics [20], investigation of environmental contaminants [21] and toxins [22], but so far not for detection and identification of acyl-CoAs.

The currently available methods for the identification of acyl-CoAs rely on full scan and subsequent ddMS^2^ measurement of all ions present in the matrix. In this study, we have developed and optimised two methods to selectively filter all acyl-CoAs from the complex biological matrices by using their characteristic fragment ions, neutral loss (M-507) and also *m*/*z* 428 in order to trigger a qualifying ddMS^2^ scan. One of these approaches which is based on in-source fragmentation ideally detects only acyl-CoAs in the corresponding ddMS^2^ experiment and avoids unnecessary ddMS^2^ scans of ions from the matrix without any inclusion list of expected compounds. To further test our methods, we performed measurements on cell extracts of the betaproteobacterium strain HxN1, affiliating with the newly described genus *Aromatoleum* [23], grown anaerobically with hexanoate and harvested in two different growth phases.

## Materials and methods

### General

Preparative column chromatography was carried out using Merck SiO_2_ (35–70 μm, type 60 Å) with *n*-hexane and ethyl acetate as eluents. ^1^H and ^13^C NMR spectra were recorded on a Bruker Avance 300 instrument. IR spectra were recorded on a Bruker Tensor 27 spectrometer equipped with a diamond ATR unit. MS and HRMS spectra were obtained with an Orbitrap Fusion tribrid mass spectrometer (Thermo Fisher Scientific, San Jose, CA, USA). HPLC purification of reference standards was done using a VWR-Hitachi LaChrom Elite HPLC and a VWR-Hitachi LaChrom Elite Diode Array Detector L-2455 (Hitachi, Tokyo, Japan) operating at 190–400 nm either with a Gemini-NX column (250 × 21.2 mm, 5-μm pore size, Phenomenex, Torrance, CA, USA) or with a Pursuit PFP column (250 × 10 mm, 5-μm pore size, Agilent Technologies, Santa Clara, CA, USA) using 10 mM ammonium formate (pH 8.1) and acetonitrile (ACN) as eluents. For sample centrifugation, a 4k10 centrifuge (Sigma, Osterode am Harz, Germany) equipped with a 12,167 fixed-angle rotor was used. Ultrapure water (Millipore, 18.2-MΩ resistivity) was used throughout the experiments. All chemicals were purchased from Sigma-Aldrich (Taufkirchen, Germany) except for coenzyme A trilithium salt, which was purchased from CoALA Bioscience (Austin, TX, USA), *trans*-2-hexenoic acid, 3-hydroxypropionic acid (30% in water) and methyl 3-oxohexanoate, which were purchased from TCI (Eschborn, Germany).

### Liquid chromatography

Chromatographic separation was performed on a Vanquish Flex UHPLC system (Thermo Fisher Scientific), equipped with a Gemini C18 column (150 × 2.0 mm, 3-μm pore size, Phenomenex) at 35 °C at an eluent flow rate of 0.4 mL/min. The amount of injection was 10 μL. Eluent A was 10 mM ammonium formate at pH 8.1 and eluent B was acetonitrile. Equilibration time was 1 min, using 100% A. The gradient was 0 to 2 min: 100% A; 2 to 23 min: 100% to 76.5% A; 23 to 26 min: 76.5% to 0% A; 26 to 29 min: 0% A; 29 to 32 min: 0% to 100% A; 32 to 42 min: 100% A.

### Mass spectrometry

Mass spectra were acquired on an Orbitrap Fusion mass spectrometer equipped with an EASY-Max NG ion source (Thermo Fisher Scientific). The ESI+ mode was used under the following heated electrospray ionisation (HESI) source parameters: the sheath gas flow rate and the aux gas flow rate were set at 40 and 10 arbitrary units, respectively; the spray voltage was 3.5 kV; the ion transfer tube temperature and the vaporiser temperature were 320 and 350 °C, respectively. The full scan measurements were performed at three different Orbitrap resolution values (60,000, 120,000 and 500,000 at *m*/*z* 200, FWHM) on acyl-CoA standards and on a biological sample, which had no influence on the mass accuracy. The full scan experiments were performed from 300 to 1000 *m*/*z* and the resolution was chosen to be 60,000. The S-lens RF level was set at 50 and the AGC target to 1e4. The maximum injection time was set to 50 ms. For the in-source fragmentation method (see below), the fragmentation energy was set to 50 V. For the ddMS^2^ mode, the resolution was chosen to be 60,000, and the isolation window was set at 1.0 *m*/*z*. At this resolution, the mass accuracy of the investigated [M+H-506.9952]^+^ monoisotopic ions (*m*/*z* up to 500) was below 1 ppm for all detected compounds (Table S1). HCD collision energy was fixed and set to 20, except for the ddMS^2^ screening method where it was set to 17; AGC target was set to 1e4. The maximum injected time was set to 118 ms. All parameters of the fragmentation cell applied in tMS^2^ were the same as in ddMS^2^ acquisition. For the calculation of elemental compositions, we set the number of phosphorus, nitrogen and sulphur atoms to three, seven and one, respectively, while the numbers of all other atoms (C, H and O) were variable. We were also able to distinguish M+2 isotopes (^34^S, ^18^O and ^13^C_2_) at the resolution of 500,000, which served as an additional criterion for the characterisation of synthetic acyl-CoA standards [24].

### Cultivation of bacterial strain

#### Sample preparation

The betaproteobacterium “*Aromatoleum*” sp. HxN1 has been subcultured in our laboratory since its isolation [25]. Cultivation was carried out in defined, bicarbonate-buffered medium, essentially as described earlier [26]. Cultures were grown in stopper-sealed flat glass bottles (500 mL) containing 400 mL medium under an anoxic atmosphere (N_2_/CO_2_ 90:10, v/v). Sodium *n*-hexanoate from a sterile stock solution was added to cultures at a final concentration of 3 mM. Growth was monitored by measuring the optical density at 600 nm (OD_600_), using a UVmini-1240 (Shimadzu, Duisburg, Germany). Cultures were harvested at ½ODmax (~ OD_600_ = 0.3) and OD_max_, taking care to avoid exposure to oxygen. Cultures were transferred into N_2_-preflushed centrifuge beakers under a steady stream of N_2_. Following centrifugation (17,000×*g*, 15 min, 4 °C), the resultant pellets were resuspended in 2 mL methanol, transferred in 1-mL aliquots into cryotubes (screw-cap tube, total volume of 2 mL, containing 0.6 g of 0.1-mm glass beads and 0.4 g of 0.7-mm zirconia beads), shock-frozen in liquid nitrogen and stored at −80 °C until extraction.

#### Extraction procedure

The cryotubes were placed into the bead beater homogeniser (Fast Prep-24 5G, MP Biomedicals, Santa Ana, CA, USA). Cell lysis was conducted with 3 cycles (maximal shaking speed) in 30-s intervals with 2-min breaks. During the breaks, the tubes were stored on dry ice. The suspension was centrifuged (14,000×*g*, 10 min, 4 °C), and the supernatant was transferred to a 4-mL glass vial and evaporated under nitrogen. The extraction was repeated two more times with 0.5 mL of ice-cold 10 mM ammonium formate (pH 7), including the same centrifugation procedure in between. The extracts were combined, frozen in liquid nitrogen and lyophilised. Dry samples were resuspended in 200 μL of water and filtered through 0.22-μm cellulose acetate centrifuge filter (in 2 mL polypropylene tubes). Then, 100 μL of the extract was transferred to an HPLC vial and the rest was stored at −80 °C. All experiments were done in triplicate. Since the CoA thioesters are very unstable, the storage of the cell pellets at −80 °C and a fast extraction procedure with the cooling steps in between and the centrifugation at 4 °C are crucial in the extract preparation, in order to avoid CoA thioester degradation.

## Results and discussion

### Liquid chromatography

**Fig. 1:**
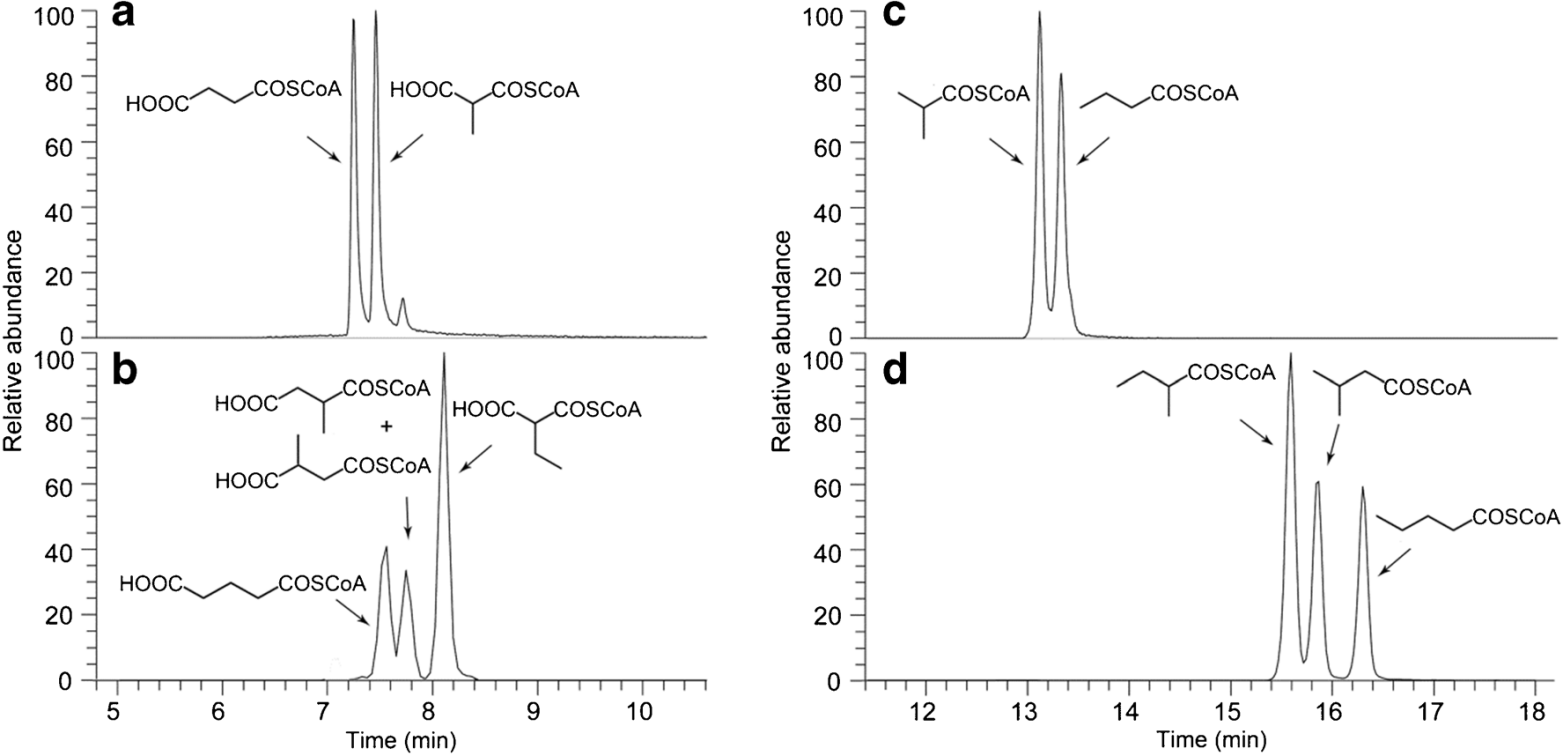
Extracted ion chromatograms of selected standard acyl-CoAs: **a** Succinyl- and methylmalonyl-CoA (*m*/*z* 361.1428), **b** glutaryl-, ethylmalonyl- and a mixture of 2- and 3-methylsuccinyl-CoA (*m*/*z* 375.1584), **c** butyryl- and isobutyryl-CoA (*m*/*z* 331.1686), **d** 2-methylbutyryl-, isopentanoyl- and pentanoyl-CoA (*m*/*z* 345.1844)

**Fig. 2:**
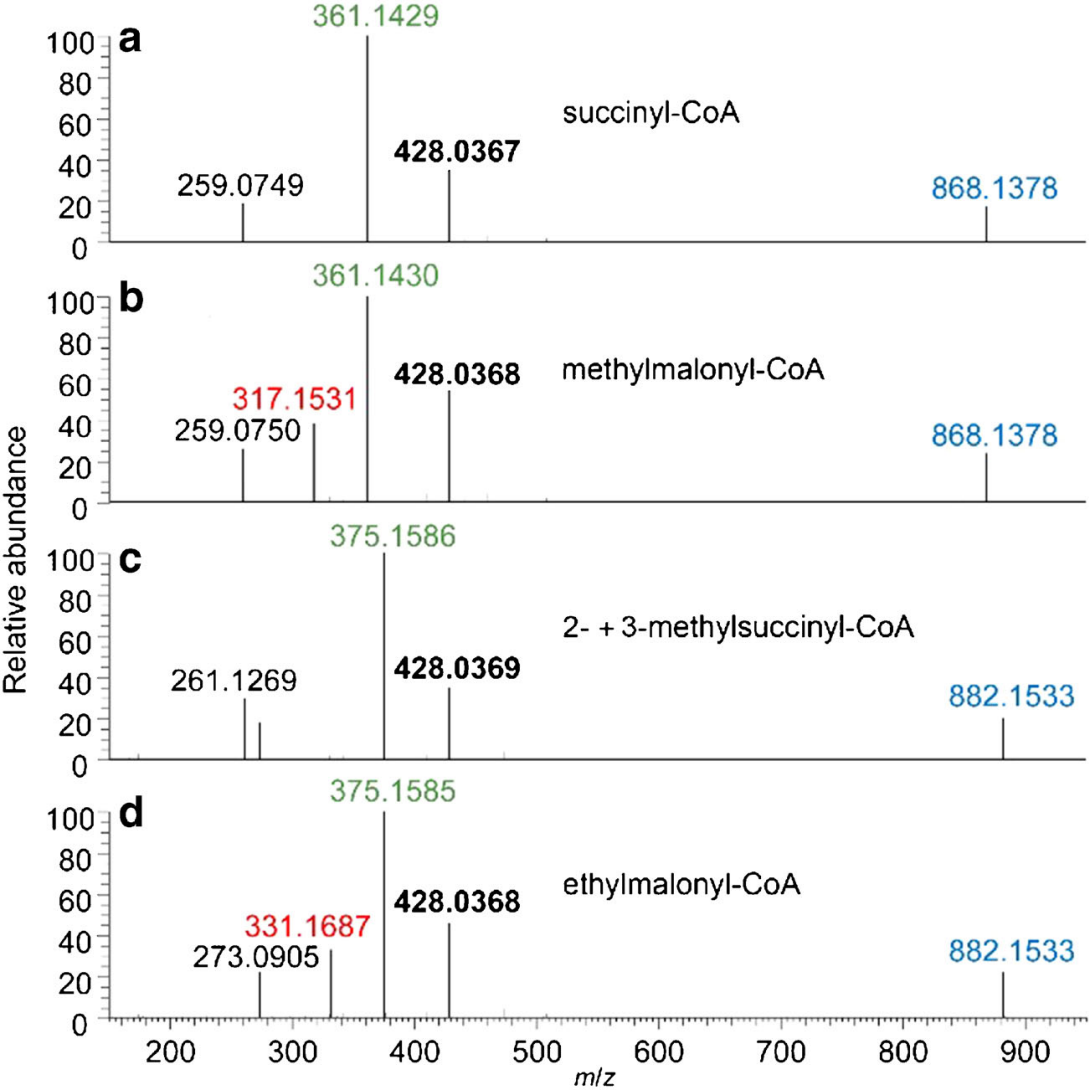
Mass spectra of CoA monothioesters of malonic and succinic acid derivatives. Blue, [M+H]^+^; green, neutral loss of 506.9952 u from [M+H]^+^; red, loss of 44 u (CO_2_) from green. Summed mass spectra of 2- and 3-methylsuccinyl-CoA are presented, since these compounds coelute (**c**). The mass spectrum of glutaryl-CoA is almost identical to the mass spectra of 2- and 3-methylsuccinyl-CoA (not shown). The ions *m*/*z* 259.0749 (**a** and **b**) and *m*/*z* 273.0905 (**d**) represent the additional loss of 102.0680 ([M-609.0632]^+^), after the neutral loss of 506.9952 which is in agreement with the previously proposed fragmentation pattern [29]

For liquid chromatographic separation of acyl-CoAs, a C_18_ reversed-phase column was used in this study agreeing with previous works on the analysis of this compound class [7, 10, 14]. It has previously been shown that slightly basic eluents are best suited for an optimal separation of acyl-CoAs [12]. Therefore, we used volatile 10 mM ammonium formate at pH 8.1 and acetonitrile as the eluting solvents, without employing ion-pairing reagents. With these settings, a good to excellent separation of various isomeric acyl-CoAs is achieved, including polar CoA monothioesters of dicarboxylic acids as well as CoA thioesters of non-polar linear and branched alkanoic acids (Fig. 1). We found that both tested isomer pairs of a malonyl- and a succinyl-CoA monothioester were baseline separated (Fig. 1a, b). The standard of methylsuccinyl-CoA used in this study was synthesised by thiolytic opening of the corresponding anhydride and thus is assumed to represent a mixture of the two possible regioisomers [27]. Furthermore, we found that glutaryl-CoA is also very well separated from the mixture of 2- and 3-methylsuccinyl-CoA (Fig. 1b). In addition, the CoA monothioesters of malonic acid derivatives are distinguishable from those of other dicarboxylic acids by mass spectrometry as reported previously [11, 28]. Namely, in the mass spectra of both malonyl-CoA derivatives, a characteristic decarboxylation fragment is observed, which is not present in the spectra of the succinyl-CoA derivatives (Fig. 2). The isobutyryl-/butyryl-CoA pair of isomers is almost baseline separated (Fig. 1c), similar to the pair of branched C_5_ monocarboxylic acid isomers that are in addition fully separated from the linear isomer (Fig. 1d). Overall, these results appear to indicate that CoA monothioesters of linear dicarboxylic acids elute earlier than those of their branched isomers, while in case of CoA thioesters of alkanoic acids, the linear isomers elute later than the branched ones, which may be helpful for the identification of unknowns.

### Mass spectrometry

**Fig. 3:**
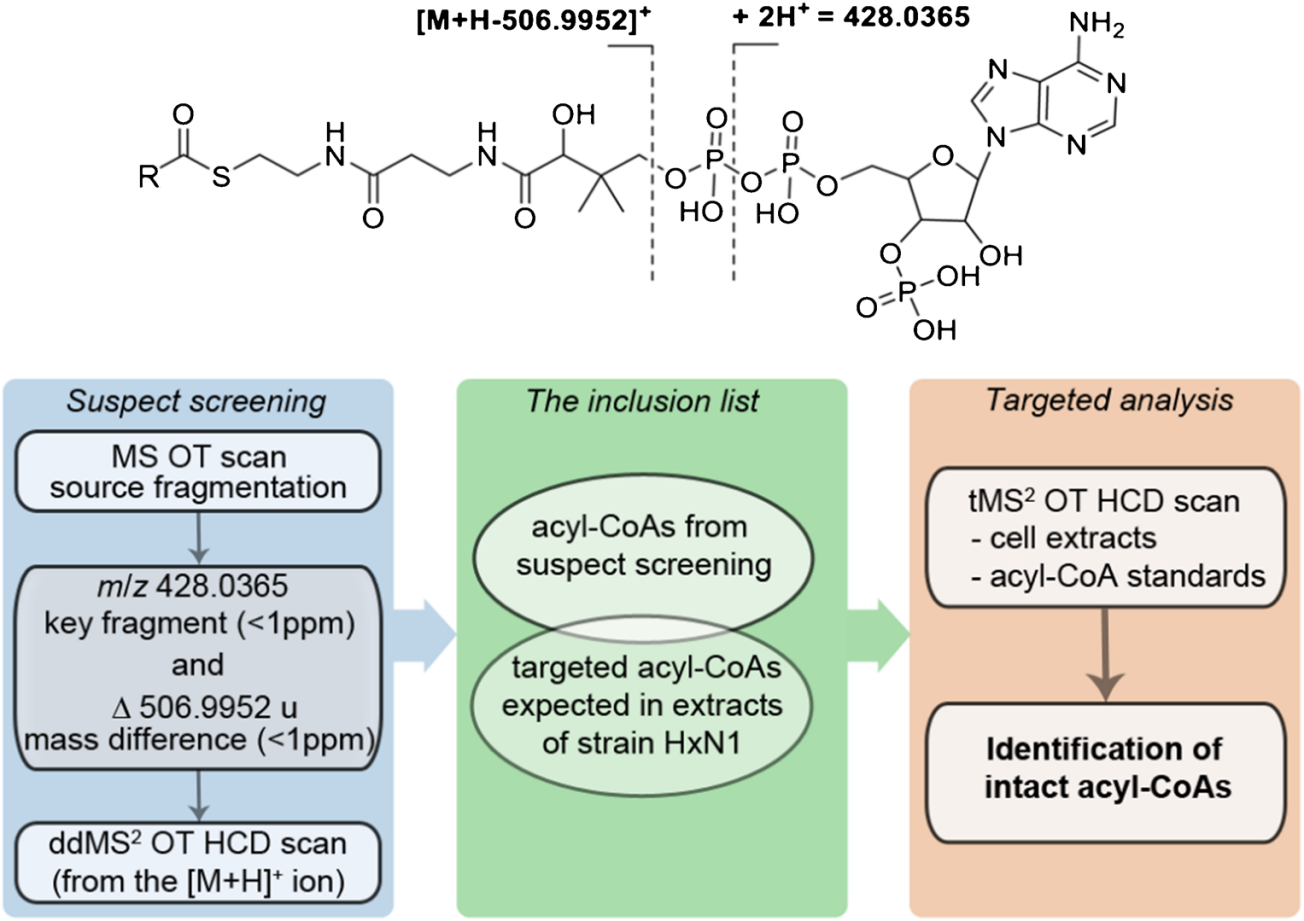
Representation of the Orbitrap Fusion ISF method developed for the analysis of CoA thioesters (below), relying on their characteristic mass spectrometric fragmentation pattern (above). MS, full scan measurement; OT, Orbitrap detection; ddMS^2^, data-dependent MS/MS; tMS^2^, targeted MS/MS

For the analysis of acyl-CoAs in biological samples, we developed two methods for suspect screening (schematically represented in Fig. S1). Both methods rely on specific fragmentation patterns of the target compounds, which are generated either by an optimised in-source fragmentation (ISF method) in a full scan Orbitrap measurement or by optimised HCD fragmentation (ddMS^2^ method). These methods provide the input data for a subsequent classical targeted analysis. A simplified schematic representation of the preferred method (ISF method), which will be explained in the following, is shown in Fig. 3.

### Suspect screening

#### In-source fragmentation (ISF) method

In order to detect acyl-CoAs in complex biological matrices selectively, we developed a method that is based on the well-defined fragmentation pattern of these compounds (Fig. 3). In contrast to the full MS/AIF (all-ion fragmentation) experiments established on various Q-Exactive mass spectrometers, in which the AIF fragmentation takes place in the HCD cell and is carried out alternately to a full MS scan, this approach is based on a discrete fragmentation of all ions in the source. For this purpose, the ionisation energy was determined in such a way that the respective molecular ion is preserved as a base peak in addition to the acyl-CoA-specific fragment ions. The usage of an optimised in-source fragmentation in combination with HRAM Orbitrap detection offers the possibility to apply a method that directly exploits the two most characteristic fragmentations as preselection criteria for a ddMS^2^ detection of acyl-CoAs. The first step includes a full scan Orbitrap measurement (MS OT) with an in-source fragmentation, which allows detecting the key fragment ion *m*/*z* 428.0365 representing the adenosine 3′,5′-diphosphate moiety and a product ion with the characteristic neutral loss of 506.9952 representing the adenosine 3′-phosphate-5′-diphosphate moiety (Fig. 3). The voltage applied for in-source fragmentation was optimised such that the energy was sufficient to induce the abovementioned fragmentations, but still allowed detection of the corresponding molecular ions as the most abundant ones (Fig. S2). Although the intensity of fragment ions depends on the structure of the acyl rest and notably is different among acyl-CoAs, the in-source fragmentation energy of 50 V gave the desired fragmentation patterns for all tested compounds. The next step in the assembled method is a *targeted mass trigger*, which in our case is the already mentioned *m*/*z* 428.0365 with a mass tolerance of <1 ppm. This means that if the key fragment is detected, the further ion selection should be performed according to the following conditions. The first is a filter for the user-defined *precursor selection range* followed by an *intensity* filter. The latter has been tested with two different values (2e3 and 1e4) not only on standard compounds, but also on a test sample to ensure method utility for biological samples. Since no significant difference in the patterns of the detected acyl-CoAs was observed between these two settings, and considering that a low value of intensity threshold can trigger matrix ions, we set this filter at 1e4 for all further experiments. The next filter applied was *dynamic exclusion*, which gives an opportunity to narrow a time range of ion detection as well as to limit the number of scan repetitions and subsequently ensures a detection of possibly coeluting compounds. The last screening filter was *targeted mass difference*. It identifies all pairs of ions in the spectrum that have the exact mass difference of 506.9952 u within a predefined mass tolerance of <1 ppm. In addition, it offers the opportunity to perform subsequent ddMS^2^ on the highest *m*/*z* ion in the pair that is expected to be the precursor ion for the neutral loss of 506.9952 u. The whole method is designed in a way to preselect all compounds (ideally only acyl-CoAs), which exhibit the key fragment ion as well as the exact neutral loss, and subsequently to perform MS^2^ acquisition on the corresponding molecular ion. Only if all mentioned conditions are met, the corresponding molecular ions are selected and sent to the ion routing multipole for HCD fragmentation. The subsequent MS^2^ spectra were generated in the Orbitrap to confirm the fragmentation pattern and accurate masses of the molecular ions. Overall, the procedure described results in a list of detected acyl-CoAs arranged according to increasing molecular weight, which then is used as input for the targeted analysis. The feasibility of the approach described was verified using a mixture of eleven commercially available acyl-CoA standards, which were all unambiguously and correctly detected in the suspect screening (Table 1). Subsequent application of the method to the samples of the bacterial cultures revealed the presence of 35 different acyl-CoAs in total (Table 1), which will be discussed in more detail below. Notably, all criteria that trigger the ddMS^2^ scan (neutral loss and target mass) could be determined in a survey scan with a mass accuracy <1 ppm over wide dynamic range of four orders of magnitude.

**Table 1:** CoA thioesters detected in the extracts of strain HxN1 (inclusion list for the targeted analysis)

No.	Compound	Formula [M+H]^+^	½OD_max_	OD_max_
	Identified acyl-CoAs			
1	3-Hydroxypropionyl-CoA^b^	C_24_H_41_N_7_O_18_P_3_S	+	+
2	Malonyl-CoA^**a**^	C_24_H_39_N_7_O_19_P_3_S	+	+
3	Succinyl-CoA^**a**^	C_25_H_41_N_7_O_19_P_3_S	+	+
4	Methylmalonyl-CoA^**a**^	C_25_H_41_N_7_O_19_P_3_S	+	+
5	Glutaryl-CoA^**a**^	C_26_H_43_N_7_O_19_P_3_S	+	+
6	Methylsuccinyl-CoA^b^	C_26_H_43_N_7_O_19_P_3_S	+	−
7	Ethylmalonyl-CoA^b^	C_26_H_43_N_7_O_19_P_3_S	+	+
8	**Acetyl-CoA**^**a**^	**C**_**23**_**H**_**39**_**N**_**7**_**O**_**17**_**P**_**3**_**S**	+	+
9	**Acetoacetyl-CoA**^**a**^	**C**_**25**_**H**_**41**_**N**_**7**_**O**_**18**_**P**_**3**_**S**	+	+
10	**3-Hydroxybutyryl-CoA**^**a**^	**C**_**25**_**H**_**43**_**N**_**7**_**O**_**18**_**P**_**3**_**S**	+	+
11	3-Hydroxy-3-methylbutyryl-CoA^b^	C_26_H_45_N_7_O_18_P_3_S	+	+
12	Propionyl-CoA^**a**^	C_24_H_41_N_7_O_17_P_3_S	+	+
13	**Crotonyl-CoA**^a^	**C**_**25**_**H**_**41**_**N**_**7**_**O**_**17**_**P**_**3**_**S**	+	+
14	Isobutyryl-CoA^**a**^	C_25_H_43_N_7_O_17_P_3_S	+	+
15	**3-Oxohexanoyl-CoA**^b^	**C**_**27**_**H**_**45**_**N**_**7**_**O**_**18**_**P**_**3**_**S**	+	+
16	**Butyryl-CoA**^**a**^	**C**_**25**_**H**_**43**_**N**_**7**_**O**_**17**_**P**_**3**_**S**	+	+
17	**3-Hydroxyhexanoyl-CoA**^b^	**C**_**27**_**H**_**47**_**N**_**7**_**O**_**18**_**P**_**3**_**S**	+	−
18	2-Methylbutyryl-CoA^b^	C_26_H_45_N_7_O_17_P_3_S	+	−
19	Isopentanoyl-CoA^b^	C_26_H_45_N_7_O_17_P_3_S	+	−
20	Pentanoyl-CoA^b^	C_26_H_45_N_7_O_17_P_3_S	+	+
21	Benzoyl-CoA^b^	C_28_H_41_N_7_O_17_P_3_S	+	+
22	Phenylacetyl-CoA^b^	C_29_H_43_N_7_O_17_P_3_S	+	+
23	***trans*-2-Hexenoyl-CoA**^**b**^	**C**_**27**_**H**_**45**_**N**_**7**_**O**_**17**_**P**_**3**_**S**	+	+
24	**Hexanoyl-CoA**^**b**^	**C**_**27**_**H**_**47**_**N**_**7**_**O**_**17**_**P**_**3**_**S**	+	+
	Tentatively identified acyl-CoAs			
25	Mesaconyl-CoA	C_26_H_41_N_7_O_19_P_3_S	+	+
26	3-Methylmalyl-CoA	C_26_H_43_N_7_O_20_P_3_S	+	+
	Unidentified acyl-CoAs			
27	Unknown-1	C_27_H_45_N_7_O_17_P_3_S	+	+
28	Unknown-2	C_27_H_45_N_7_O_17_P_3_S	+	+
29	Unknown-3	C_26_H_45_N_7_O_18_P_3_S	+	−
30	Unknown-4	C_27_H_45_N_7_O_19_P_3_S	+	+
31	Unknown-5	C_28_H_49_N_7_O_18_P_3_S	−	+
32	Unknown-6	C_29_H_43_N_7_O_18_P_3_S	+	−
33	Unknown-7	C_27_H_43_N_7_O_20_P_3_S	+	−
34	Unknown-8	C_28_H_47_N_7_O_19_P_3_S	+	−
35	Unknown-9	C_31_H_53_N_7_O_20_P_3_S	+	−

^a^Confirmed by comparison with a commercially available reference standard

^b^Confirmed by comparison with a reference standard synthesised in this study

**Fig. 4:**
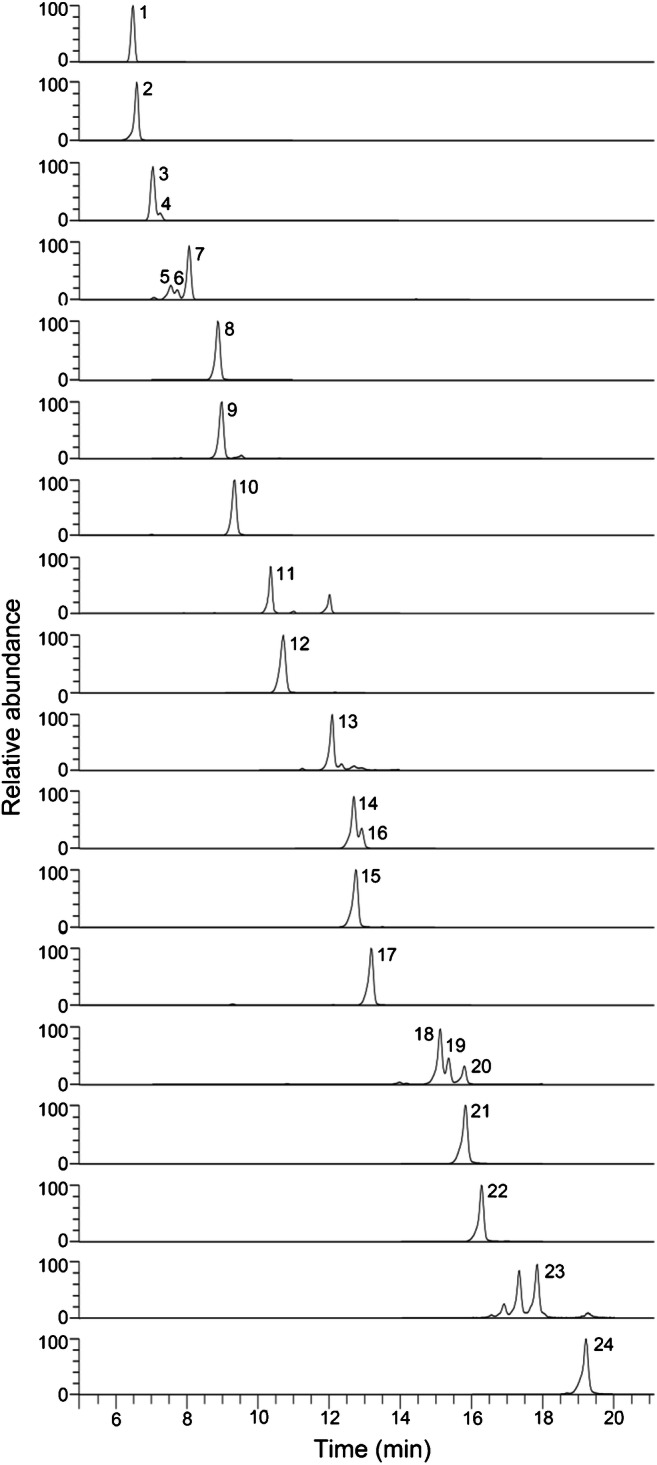
Extracted ion chromatograms of all acyl-CoAs identified in cells of strain HxN1 by standard comparison. The accurate mass of the product ion after neutral loss was used as the extraction mass (Table S1). Corresponding peak annotations are given in Table 1

No., compounds numbering corresponds to their elution order (shown in Fig. 4)

Acyl-CoA intermediates of the hexanoate degradation pathway are highlighted

½OD_max_ and OD_max_ indicate the time point of harvesting strain HxN1 for analysis of acyl-CoAs

#### Data-dependent MS^2^ (ddMS^2^) method

This method was also designed to perform an intelligent MS^2^ triggering of all relevant target ions, like in the ISF method, using the same instrument parameters but without in-source fragmentation. In this screening method, the full MS OT survey scan was followed by intensity and dynamic exclusion filters (with the same parameters like in the ISF method) and by serial ddMS^2^ OT HCD scans with quadrupole isolation of all precursor ions in a selection range from *m*/*z* 810 to 1000. Before this acquisition, the acyl-CoA fragmentation was optimised in both HCD and CID cells (Fig. S3 and S4). Ultimately, HCD fragmentation was preferred at 17% because it mainly provides the substance class specific acyl-CoA fragment ions. Then, the previously described targeted mass trigger and mass difference filter were defined, in order to ideally trigger only molecular ions of acyl-CoA species in the subsequent ddMS^2^ scan. Considering the application of two consecutive ddMS^2^ scans, this method could be considered as a “pseudo” neutral loss method that mimics the traditional neutral loss method in a triple quadrupole mass spectrometer (Fig. S1).

#### Methods comparison

In order to compare the efficiency of the methods described, mixtures of 32 acyl-CoA standard compounds at five different concentrations (Table S2) were measured. Only in the case of diluted samples, some differences could be observed. Namely, six compounds were detected using the ISF method but not using the ddMS^2^ method at low concentrations. Contrarily, only one compound was detected using the ddMS^2^ method but not using the ISF method. We further compared the methods by analysing biological samples, which were not diluted but for which different amounts were injected (Table S3). Similar to the results from the standard comparison, only at lower abundance of the acyl-CoAs, some differences could be observed. In nine cases, compounds were detected using the ISF method but not using the ddMS^2^ method. On the other hand, only 3-hydroxypropionyl-CoA was detected using the ddMS^2^ method but not using the ISF method. Overall, the ISF method showed better results at low concentrations of the acyl-CoAs. The limit of detection for the ISF method was investigated by analysing a dilution series of standards (compounds marked with “a” in Table 1). These measurements revealed that in general 200 fMol of acyl-CoA is required to trigger the ddMS^2^ experiment with an even lower limit of 100 fMol for certain acyl-CoAs, namely 3-hydroxybutyryl-CoA, methylmalonyl-CoA and glutaryl-CoA.

The high resolution of the mass spectrometer and mass error constant <1 ppm over an extraordinary dynamic range enable the exact calculation of mass differences (neutral loss of the corresponding ion pair) after non-selective in-source fragmentation of all ions. That allows precise and selective identification of the acyl-CoAs already in the survey scan even in the case of a high background. These results documented that the ISF method was very effective for the suspect screening of acyl-CoAs in all investigated samples. The methods described here are not directly comparable with previously published approaches, which were not specifically developed for the analysis of acyl-CoAs. In particular, previous reports predominantly described targeted methods, while our work focuses on non-targeted screening. Nevertheless, our approaches allow simultaneous detection of acyl-CoAs with different acyl rests, such as benzoyl-CoA and phenylacetyl-CoA along with CoA thioesters from fatty acid metabolism.

#### Targeted analysis

As the result of the suspect screening, a list (hereafter named **inclusion list**, Table 1) of acyl-CoAs detected in a sample is created. A compound is considered as an acyl-CoA when both characteristic fragments are present in the ddMS^2^ spectrum and the [M+H-506.9952]^+^ ion is the most abundant, followed by the ion *m*/*z* 428.0365. The same pattern was observed for all analysed acyl-CoAs, including the reference standards, independent of the structure of the acyl rest. Importantly, we have not observed any fragment ions resulting from the acyl rest in agreement with all previously published data [2, 7, 10, 28], with the only exception being the decarboxylation of malonyl-CoA derivatives mentioned above. The next very important criterion is the mass accuracy of the ions [M+H]^+^ and [M+H-506.9952]^+^. Only compounds within a deviation below 1 ppm are being taken into consideration. All compounds with odd *m*/*z* values of the [M+H]^+^ ion from the suspect screening list are neglected. This list is amended by acyl-CoAs, which are expected to occur in a given biological sample considering the available information on the metabolic processes of interest in combination with the applied cultivation condition(s), in particular the substrate provided. For the biological samples used in this study, all acyl-CoAs targeted in this sense (see below) were already detected by the suspect screening and thus incorporated in the inclusion list.

The comprehensive inclusion list is then used for the targeted analysis (tMS^2^). This list includes the start and end times for each target compound, which are adjusted according to the corresponding retention times of reference standards or retention times of acyl-CoAs detected during the suspect screening, respectively. The main aim is to confirm the presence and identity of acyl-CoAs detected in the suspect screening, including the “expected” acyl-CoAs, and to compare their retention behaviours and fragmentation patterns with those of reference standards (synthetic and commercial). The list of all standards, their accurate mass deviations and relative intensity (acetyl-CoA is used as a reference signal) of identified compounds in both biological samples are shown in Table S1. The accurate mass deviations of molecular ions are in the range from −0.81 ppm to +0.69 ppm. It is worth mentioning that the signal intensities of different acyl-CoAs covered a dynamic range of 10^4^ (shown in Table S1 (relative intensities) and in Fig. S5). The application of this procedure to biological samples is described in the next section.

### Identification of acyl-CoAs in strain HxN1

**Fig. 5:**
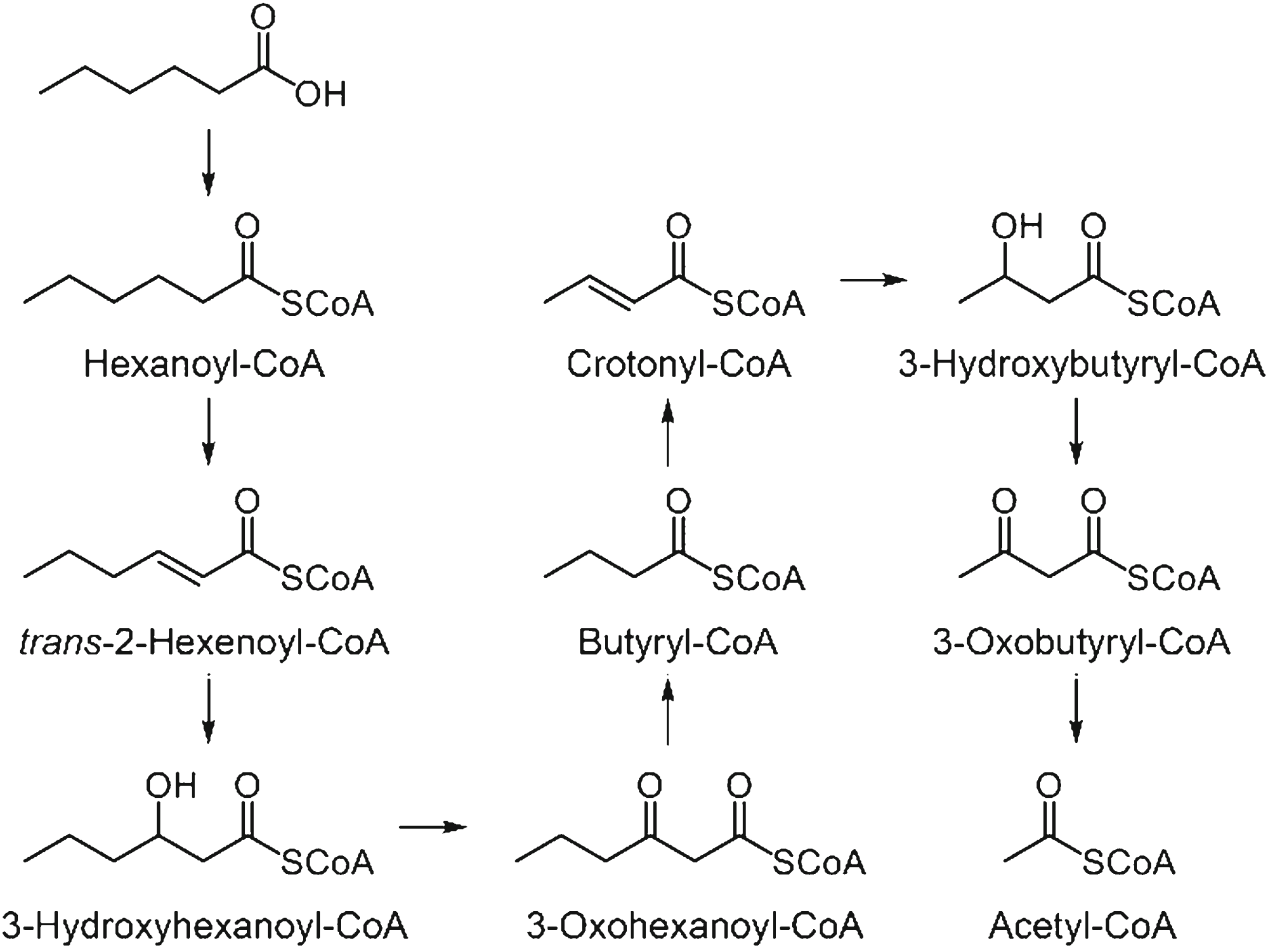
Pathway of degradation of hexanoic acid to acetyl-CoA in strain HxN1. The enzymatic reactions leading from 3-oxohexanoyl-CoA to butyryl-CoA and acetoacetyl-CoA to acetyl-CoA, respectively, go along with the additional formation of a molecule of acetyl-CoA in both cases, such that overall one molecule of hexanoic acid is transformed to three molecules of acetyl-CoA

In total, 35 different acyl-CoAs were detected across the two samples, with 34 at ½OD_max_ and 26 at OD_max_ (Table 1, according to their elution order). By comparison with reference standards, 24 of them were unambiguously identified. Out of these, eleven were commercially available, while the remaining thirteen have been synthesised within the framework of this study using standard procedures (see Supplementary Information for details). The extracted ion chromatograms of all identified acyl-CoAs are depicted in Fig. 4. All nine targeted acyl-CoAs from the degradation pathway of hexanoate (Fig. 5) were clearly identified in the sample retrieved at ½OD_max_, representing active growth of strain HxN1. In contrast, 3-hydroxyhexanoyl-CoA was missing in the OD_max_ sample, indicating that its concentration was below detection limit in the stationary growth phase. The relative intensities of all identified acyl-CoAs in both samples are presented in Table S1. The amount of hexanoyl-CoA is significantly lower in the stationary than in the linear growth phase by two orders of magnitude. Additionally, the relative amounts of 3-oxohexanoyl-CoA and crotonyl-CoA decreased approximately one order of magnitude in the stationary growth phase. These observations are likely related to markedly different metabolic states of the cells during these two fundamentally different growth phases. Alongside the expected intermediates of the hexanoate degradation pathway, almost all metabolites occurring in the ethylmalonyl-CoA pathway were also detected in both samples [30]. While crotonyl-CoA, 3-hydroxybutyryl-CoA, acetoacetyl-CoA and acetyl-CoA may occur in both pathways, propionyl-CoA, methylmalonyl-CoA, succinyl-CoA, mesaconyl-CoA and 3-methylmalyl-CoA would be exclusive intermediates of the ethylmalonyl-CoA pathway. Mesaconyl-CoA and 3-methylmalyl-CoA have only tentatively been identified according to their accurate molecular masses and by comparing their retention times with that of the methylsuccinyl-CoA standard, which elutes approximately 0.5 and 0.3 min before them, respectively. The conversion of crotonyl-CoA to ethylmalonyl-CoA has been reported to occur in other bacteria [31]. Furthermore, carboxylation of butyryl-CoA to ethylmalonyl-CoA has been reported to occur in an alternative route for carbon assimilation rather than through the glyoxylate pathway [32]. Since the intermediates of both pathways are detected in the cells of strain HxN1, the role of ethylmalonyl-CoA is not fully clear. Additionally, propionyl-CoA could be a product of more than one metabolic process, including the 3-hydroxypropionyl-CoA pathway, but since acryloyl-CoA is missing in the samples, it is difficult to attribute propionyl-CoA to a particular origin [33].

Additionally, acyl-CoAs possibly related to the degradation of branched and aromatic amino acids are present in the samples. For example, isobutyryl-CoA, 2-methylbutyryl-CoA and isovaleryl-CoA could derive from valine, isoleucine and leucine, respectively [34]. In the same degradation pathways, 3-hydroxy-2-methylbutyryl-CoA (3H2MBCoA) has also been reported as an intermediate [34]. Since we detected a compound with the same accurate mass as 3H2MBCoA, we synthesised it as well as the isomeric 3-hydroxy-3-methylbutyryl-CoA (3H3MBCoA). After standard comparison, it was clear that the latter isomer was present in our sample. The role of this metabolite in strain HxN1 is so far unclear. It has been shown that 3H3MBCoA is involved in the biosynthesis of D-anthrose [35] and also is an intermediate in the leucine degradation pathway in humans [36]. Additionally, in strain HxN1, possible intermediates of the phenylalanine degradation pathway have been identified, such as phenylacetyl-CoA, benzoyl-CoA and glutaryl-CoA. The latter is a part of the central benzoyl-CoA pathway of anaerobic degradation of monoaromatic compounds [37].

Unfortunately, a number of detected acyl-CoAs remained unidentified. According to the accurate masses, unknown-1 and unknown-2 represent structural isomers of hexenoyl-CoA and since they elute before it (Fig. 4), they could be branched C_6_ derivatives, which is in agreement with the eluting order of CoA thioesters of linear and branched alkanoic acids, as discussed before (section “Liquid chromatography”). Likewise, unknown-3, which elutes after 3H3MBCoA and 3H2MBCoA, could be for example 3-hydroxypentanoyl-CoA (Fig. 4). Since we also detected pentanoyl-CoA in the samples, this intermediate might indicate degradation of the latter according to the pathway discussed above for hexanoate. This hypothesis can be supported by the presence of unknown-5, which could be a dihomologue of unknown-3 and would also fit to a possible β-oxidation of a longer chain metabolite. Unkown-8 has a short retention time (data not shown) and according to its accurate mass has the same formula as pimeloyl-CoA, which also is an intermediate in the central anaerobic degradation pathway of benzoyl-CoA and a precursor of glutaryl-CoA [37].

## Conclusion

The chromatographic method applied in this study provides a good separation of isomeric acyl-CoAs without using ion-pairing reagents, which is of high relevance, since the invariant CoA moiety is responsible for all characteristic fragment ions of these compounds thus making isomers widely undistinguishable by MS. Nevertheless, these specific fragmentations are instrumental for the highly selective identification of acyl-CoAs in the biological matrix. We took advantage of this feature to develop two methods for profiling of intact acyl-CoAs in bacterial cells, using a high-resolution tribrid mass spectrometer. The methods rely on two highly characteristic fragmentations as a main prefilter for the ddMS^2^ measurement of acyl-CoAs. In the method, which showed a slightly better performance, the combination of the gentle in-source fragmentation and subsequent HRAM Orbitrap detection in a survey scan made it possible to explicitly recognise key fragments over a dynamic range of four orders of magnitude at a mass accuracy < 1 ppm and to calculate ion pairs in the full scan spectrum, whose exact mass difference represents a specific neutral loss. Linking both pieces of information to define the subsequent ddMS^2^ scan of selected molecular ions led to the selective and simple detection of metabolites of this class of compounds. The combined suspect screening and targeted analysis approach described in this study could be a blueprint for developing procedures for the identification of molecules, which belong to different compound classes and which are distinguishable by characteristic fragmentation patterns, such as glycosides, glutathione conjugates, steroids and lipids.

We applied the new methods for analysis of acyl-CoAs to investigate the denitrifying betaproteobacterium “*Aromatoleum*” sp. strain HxN1 anaerobically grown with hexanoate. All acyl-CoAs expected to occur in the hexanoate degradation pathway were already detected in the suspect screening, confirming the selectivity and sensitivity of the developed methods. This study provides experimental evidence for the existence and activity of different metabolic pathways in strain HxN1 such as β-oxidation of fatty acids, amino acid degradation, ethylmalonyl-CoA and 3-hydroxypropanoyl-CoA pathways.

## Supplementary information

ESM 1 (PDF 1805 kb)

## Data Availability

The datasets generated and analysed during the current study are available from the corresponding author on reasonable request.
