# Supplementary material for: Suspect screening and targeted analysis of acyl coenzyme A thioesters in bacterial cultures using a high-resolution tribrid mass spectrometer
Source: Anal Bioanal Chem. 2021 Apr 21;413(14):3599–610. doi: 10.1007/s00216-021-03318-3 (PMC8141488; doi:10.1007/s00216-021-03318-3)
Supplement: Supplementary file 1 — (PDF 1805 kb) [file 216_2021_3318_MOESM1_ESM.pdf]

Supplementary Information

Suspect screening and targeted analysis of acyl coenzyme A thioesters in bacterial cultures using a high-resolution tribrid mass spectrometer

Nevenka Cakić[†]*, Bernd Kopke[†], Ralf Rabus[‡] and Heinz Wilkes[†]

[†]Organic Geochemistry and [‡]General & Molecular Microbiology, Institute for Chemistry and Biology of the Marine Environment (ICBM), Carl von Ossietzky University of Oldenburg, 26111 Oldenburg, Germany

*Corresponding author: nevenka.cakic@uni-oldenburg.de

Synthesis of reference standards

General method for ester hydrolysis. The corresponding ester was dissolved in 1 mL THF and 1 mL of 1 M NaOH was added. The mixture was stirred for 4 h at 40 °C and then extracted with diethyl ether (Et_2O). The water layer was acidified to pH 2 with 1 M HCl and then extracted three times with ethyl acetate (EtOAc). The organic layers were combined, dried over $MgSO_4$, filtered and evaporated.

Synthesis of CoA thioesters. For the synthesis of the majority of CoA thioesters a 1,1'-carbonyldiimidazole (CDI) method was applied.[1] Namely, the corresponding acid (1 eq, 1.3 µmol) was dissolved in 0.2 mL of ACN and cooled to 0 °C and CDI (1 eq, 1.3 µmol, 0.21 mg) dissolved in 0.1 mL ACN was added. After mixing for 1 h, a solution of CoA trilithium salt (1 eq, 1.3 µmol, 1 mg) in 0.2 mL 0.1 M $NaHCO_3$ was added and stirred further for additional 1 h at 0 °C. The corresponding CoA thioester was characterised by HRMS and tandem mass spectrometry (Table S1). All CoAs with a hydroxy group in the acyl rest were synthesised using the CDI method, without previous protection.[2] Synthesis of 3-hydroxyhexanoyl-CoA started from methyl 3-oxohexanoate, which was reduced to methyl 3-hydroxyhexanoate. The latter was then hydrolysed according to the general method described above and the free acid obtained was coupled with CoA using the CDI procedure. The synthesis of 3-oxohexanoyl-CoA was performed according to a previously published procedure starting from methyl 3-oxohexanoate.[3] For the synthesis of *trans*-2-hexenoyl-CoA a modified procedure was applied, using *N*,*N*′-diisopropylcarbodiimide (DIC, 1 eq, 1.3 µmol, 0.2 µL) instead of CDI to avoid addition of imidazole to the double bond.
For the synthesis of monothioesters of dicarboxylic acids, an anhydride method for methylsuccinyl-CoA and a transthioesterification method for ethylmalonyl-CoA were applied. In the first case, methylsuccinic acid (3 eq, 3.9 µmol, 0.5 mg) was refluxed in the presence of acetic acid anhydride (1 mL) for 2 h. After evaporation, the crude product was dissolved in 0.2 mL of ACN, mixed with a solution of CoA trilithium salt (1 eq, 1.3 µmol, 1mg) in 0.2 mL 0.1 M $NaHCO_3$ and stirred further for 1 h at 0 °C. In the second case, diethyl ethylmalonate was first hydrolysed according to the general method described above to produce ethylmalonic acid, then reacted with acetone to produce the corresponding Meldrum's acid derivate (2,2-dimethyl-5-ethyl-1,3-dioxan-4,6-dion), which was further transformed to a monothioester using 4-methoxythiophenol. The product (2-((4-methoxyphenylthio)carbonyl)butanoic acid; 1 eq, 1.3 µmol, 0.3 mg) was dissolved in 0.2 mL ACN and cooled to 0 °C, then a solution of CoA trilithium salt (1 eq, 1.3 µmol, 1 mg) in 0.2 mL 0.1 M $NaHCO_3$ was added, and the mixture was stirred for 1 h at 0 °C and an additional 1 h at ambient temperature.

Methyl 3-hydroxyhexanoate. Methyl 3-oxohexanoate (1 eq, 1.5 mmol, 0.2 g) was dissolved in MeOH (5 mL) and cooled to 0°C in an ice–water bath, then $NaBH_4$ (1.8 mmol, 1.2 eq) was added to the solution. After stirring the mixture for 2 h at ambient temperature, water (10 mL) was added, and the product was extracted three times with EtOAc. The organic layers were combined, dried over $MgSO_4$ and evaporated after filtration. The residue was purified by column chromatography using 40% EtOAc in *n*-hexane to obtain the title compound (0.13 g, 65 %). ^1H NMR (300 MHz, $CDCl_3$): δ 0.86 (3H, t, J = 6.9 Hz), 1.25-1.48 (4H, m), 2.34 (1H, dd, J_1 = 16.5 Hz, J_2 = 8.7 Hz), 2.44 (1H, dd, J_1 = 16.5 Hz, J_2 = 3.3 Hz), 2.86 (1H, br s), 3.64 (3H, s), 3.91-3.99 (1H, m). ^{13}C NMR (75 MHz, $CDCl_3$): δ 13.9, 18.7, 38.6, 41.1, 51.7, 67.7, 173.5. IR (ATR): nu(tilde) 3464 (s, br), 2959 (s), 2934 (s), 2874 (m), 1724 (vs), 1458 (m), 1437 (s), 1368 (w), 1307 (w), 1294 (w), 1262 (m), 1167 (s), 1122 (m), 1075 (m), 1047 (m), 1020 (s), 994 (s), 848 (m), 745 (w), 615 (w). HRMS (ESI+) calculated for $[C_7H_{14}O_3Na]^+$: 169.0835, found 169.0840.

3-Hydroxyhexanoic acid. The compound was obtained according to the general procedure for ester hydrolysis describe above. For the synthesis, 0.1 g of starting material was used to obtain 0.07 g of product (78%). ^{1}H NMR (300 MHz, $CDCl_3$): δ 0.86 (3H, t, J = 6.9 Hz), 1.25-1.52 (4H, m), 2.39 (1H, dd, J_1 = 16.5 Hz, J_2 = 9.0 Hz), 2.49 (1H, dd, J_1 = 16.5 Hz, J_2 = 3.3 Hz), 3.94-4.09 (1H, m), 7.02 (1H, br s). ^{13}C NMR (75 MHz, $CDCl_3$): δ 13.9, 18.6, 38.5, 41.1, 67.8, 177.8. IR (ATR): nu(tilde) 3445 (s, br), 2960 (s), 2934 (s), 2874 (m), 2590 (w), 1708 (vs), 1467 (m), 1410 (m), 1382 (m), 1294 (m), 1262 (m), 1224 (m), 1174 (s), 1122 (m), 1075 (m), 1044 (m), 1017 (m), 968 (w), 908 (m), 881 (m), 847 (m), 731 (s). HRMS (ESI+) calculated for $[C_6H_{12}O_3Na]^+$: 155.0684, found 155.0679.

Methyl 2-(2-propyl-1,3-dioxolan-2-yl)acetate. The compound was synthesised according to a previously published method.[3] The chromatographic purification was achieved with 6% EtOAc in *n*-hexane. For the synthesis, 0.5 g of methyl 3-oxohexanoate was used to obtain 0.35 g of product (54%). ^{1}H NMR (300 MHz, $CDCl_3$) δ 0.86 (3H, t, J = 7.2 Hz), 1.31-1.39 (2H, m), 1.68-1.74 (2H, m), 2.59 (2H, s), 3.62 (3H, s), 3.88-3.94 (4H, m). ^{13}C NMR (75 MHz, $CDCl_3$) δ 14.2, 16.8, 39.9, 42.5, 51.7, 65.1, 109.3, 170.0. IR (ATR): nu(tilde) 2960 (s), 2876 (m), 1735 (vs), 1467 (m), 1455 (m), 1437 (s), 1352 (m), 1331 (m), 1295 (m), 1245 (m), 1212 (s), 1127 (s), 1070 (s), 1038 (s), 1024 (s), 967 (s), 948 (s), 892 (w), 837 (s), 767 (w), 741 (w), 688 (w), 545 (w). HRMS (ESI+) calculated for $[C_9H_{16}O_4Na]^+$: 211.0946, found 211.0941.

2-(2-Propyl-1,3-dioxolan-2-yl)acetic acid. The compound was obtained according to the general procedure for ester hydrolysis described above. For the synthesis, 0.1 g of starting material was used to obtain 0.04 g of product (44%). ^{1}H NMR (300 MHz, $CDCl_3$) δ 0.94 (3H, t, J = 7.2 Hz), 1.40-1.47 (2H, m), 1.77-1.83 (2H, m), 2.71 (2H, s), 3.99-4.05 (4H, m). ^{13}C NMR (75 MHz, $CDCl_3$) δ 14.1, 16.8, 39.8, 42.4, 65.1, 109.2, 174.8. IR (ATR): nu(tilde) 2961 (s), 2935 (s), 2875 (s), 1710 (vs), 1467 (m), 1457 (m), 1435 (m), 1405 (m), 1375 (m), 1297 (m), 1247 (m), 1211 (m), 1134 (m), 1067 (s), 1037 (s), 1020 (s), 972 (m), 950 (s), 882 (w), 838 (s), 801 (w), 755 (w), 732 (w), 642 (m). HRMS (ESI+) calculated for $[C_8H_{14}O_4Na]^+$: 197.0790, found 197.0785.

Ethylmalonic acid. Diethyl ethylmalonate (9.4 mL, 50 mmol) was hydrolysed according to the general method for ester hydrolysis described above to give the title compound (4.6 g, 70%). ^{1}H NMR (300 MHz, D_2O) δ 0.79 (3H, t, J = 7.5 Hz), 1.72 (2H, p, J = 7.5 Hz), 3.26 (1H, t, J = 7.5 Hz). ^{13}C NMR (75 MHz, D_2O) 10.8, 21.9, 53.2, 173.8. IR (ATR): nu(tilde) 2981 (m), 2954 (m), 2885 (m), 2588 (m), 2524 (m), 1694 (vs), 1457 (m), 1417 (s), 1327 (m), 1298 (s), 1268 (s), 1230 (m), 1200 (s), 1091 (s), 1044 (m), 908 (s), 781 (s), 674 (s), 581 (s). HRMS (ESI+) calculated for $[C_5H_8O_4Na]^+$: 155.0320, found 155.0316.

2,2-Dimethyl-5-ethyl-1,3-dioxan-4,6-dion (Meldrum's acid derivative). Ethylmalonic acid (1 eq, 3 g, 22.7 mmol), acetic acid anhydride (1.3 eq, 29.5 mmol, 2.8 mL) and 0.1 mL of cc H_2SO_4 were mixed at 0 °C and stirred for 30 min. Then, acetone (1.4 eq, 31.8 mmol, 2.3 mL) was added and the mixture was further stirred for additional 3 h at ambient temperature. The mixture was placed at −20 °C for 18 h. The precipitate was washed with ice-cold water, dissolved in EtOAc, then dried with $MgSO_4$ and evaporated to give the title compound (3.29 g, 84%). ^{1}H NMR (300 MHz, $CDCl_3$) δ 0.98 (3H, t, J = 7.3 Hz), 1.69 (3H, s), 1.73 (3H, s), 2.10 (2H, qd, J_1 = 7.3 Hz, J_2 = 4.8 Hz), 3.47 (1H, t, J = 4.8 Hz). ^{13}C NMR (75 MHz, $CDCl_3$) δ 10.7, 20.1, 27.0, 28.4, 47.1, 104.8, 165.5. IR (ATR): nu(tilde) 2984 (m), 2944 (m), 2890 (s), 1775 (s), 1731 (vs), 1458 (m), 1438 (s), 1395 (s), 1385 (s), 1362 (w), 1347 (s), 1314 (s), 1261 (m), 1242 (m), 1201 (s), 1121 (m), 1092 (s), 1065 (s), 1001 (m), 980 (s), 918 (m), 898 (w), 874 (s), 844 (s), 778 (w), 684 (s), 630 (s), 592 (w). HRMS (ESI+) calculated for $[C_8H_{12}O_4Na]^+$: 195.0633, found 195.0629.

2-((4-Methoxyphenylthio)carbonyl)butanoic acid. The product was synthesized according to a previously published method with small modifications.[4] The corresponding Meldrum's acid derivative (1 eq, 2.9 mmol, 0.5 g) was dissolved in 3 mL of ACN under nitrogen and cooled to 0 °C. Subsequently, diisopropylethylamine (1.1 eq, 3.2 mmol, 0.56 mL) and then trimethylsilylchloride (1.2 eq, 3.48 mmol, 0.46 mL) were added. The reaction mixture was further stirred for 15 min at 0 °C, then removed from the ice-bath and 4-methoxythiophenol (1.05 eq, 3.05 mmol, 0.38 mL) was added. The mixture was heated to 45 °C and stirred for additional 12 h. After the mixture cooled down to ambient temperature, 5 mL of 0.3 M HCl was added and the water phase was extracted 3 times with 10 mL Et_2O. The organic layers were combined and washed with 1 M $NaHCO_3$ solution. The pH was adjusted to 2 using 3 M HCl and extracted three times with Et_2O. The organic phase was washed with brine, dried over $MgSO_4$ and evaporated after filtration. The product was purified by recrystallization in a toluene-chloroform solution (3:1) to provide the title compound (0.41 g, 53%). ^1H NMR (300 MHz, $CDCl_3$) 0.98 (3H, t, J = 7.2 Hz), 1.97 (2H, p, J = 7.2 Hz), 3.56 (1H, t, J = 7.2 Hz), 3.76 (3H, s), 6.88 (2H, d, J = 9.0 Hz), 7.27 (2H, d, J = 9.0 Hz). ^{13}C NMR (75 MHz, $CDCl_3$) δ 10.7, 22.6, 54.4, 59.0, 114.0, 116.3, 135.1, 160.0, 170.8, 194.1. IR (ATR): nu(tilde) 2964 (m), 2943 (m), 2880 (m), 2841 (m), 1704 (s), 1682 (s), 1591 (s), 1571 (s), 1495 (s), 1460 (s), 1441 (s), 1422 (s), 1337 (m), 1291 (m), 1251 (s), 1224 (s), 1187 (m), 1172 (m), 1107 (m), 1095 (s), 1032 (s), 980 (s), 922 (m), 860 (s), 822 (s), 802 (s), 777 (m), 661 (s), 610 (m), 542 (m). HRMS (ESI+) calculated for $[C_{12}H_{15}O_4S]^+$: 255.0686, found 255.0686.

Table S1 Acyl-CoAs identified in strain HxN1.

Compound	Formula $[M+H]^+$	Accurate mass $[M+H]^+$		Δ ppm $[M+H]^+$	Formula $[M+H-507]^+$	Accurate mass $[M+H-507]^+$		Δ ppm $[M+H-507]^+$	Relative intensity (%)	
		Exp.	Cal.			Exp.	Cal.		½OD_{max}	OD_{max}
Acetyl-CoA	$C_{23}H_{39}N_7O_{17}P_3S$	810.1329	810.1330	-0.12	$C_{13}H_{23}N_2O_4S$	303.1374	303.1373	0.33	100.00	100.00
Propionyl-CoA	$C_{24}H_{41}N_7O_{17}P_3S$	824.1487	824.1487	0.00	$C_{14}H_{25}N_2O_4S$	317.1530	317.1529	0.32	37.11	24.58
Crotonyl-CoA	$C_{25}H_{41}N_7O_{17}P_3S$	836.1484	836.1487	-0.36	$C_{15}H_{25}N_2O_4S$	329.1532	329.1529	0.91	1.47	0.10
Isobutyryl-CoA<br>Butyryl-CoA	$C_{25}H_{43}N_7O_{17}P_3S$	838.1637	838.1643	-0.72	$C_{15}H_{27}N_2O_4S$	331.1686	331.1686	0.00	155.56	71.51
3-Hydroxypropionyl-CoA	$C_{24}H_{41}N_7O_{18}P_3S$	840.1439	840.1436	0.36	$C_{14}H_{25}N_2O_5S$	333.1480	333.1479	0.30	0.05	0.02
Acetoacetyl-CoA	$C_{25}H_{41}N_7O_{18}P_3S$	852.1437	852.1436	0.12	$C_{15}H_{25}N_2O_5S$	345.1481	345.1479	0.58	0.65	0.09
2-Methylbutyryl-CoA<br>Isopentanoyl-CoA<br>Pentanoyl-CoA	$C_{26}H_{45}N_7O_{17}P_3S$	852.1796	852.1800	-0.47	$C_{16}H_{29}N_2O_4S$	345.1844	345.1842	0.58	2.10	0.31
Malonyl-CoA	$C_{24}H_{39}N_7O_{19}P_3S$	854.1231	854.1229	0.23	$C_{14}H_{23}N_2O_6S$	347.1272	347.1271	0.29	1.58	1.27
3-Hydroxybutyryl-CoA	$C_{25}H_{43}N_7O_{18}P_3S$	854.1592	854.1593	-0.12	$C_{15}H_{27}N_2O_5S$	347.1638	347.1635	0.86	5.77	0.17
trans-2-Hexenoyl-CoA	$C_{27}H_{45}N_7O_{17}P_3S$	864.1800	864.1800	0.00	$C_{17}H_{29}N_2O_4S$	357.1842	357.1842	0.00	0.60	0.08
Hexanoyl-CoA	$C_{27}H_{47}N_7O_{17}P_3S$	866.1962	866.1956	0.69	$C_{17}H_{31}N_2O_4S$	359.2001	359.1999	0.56	38.74	0.16
Succinyl-CoA<br>Methylmalonyl-CoA	$C_{25}H_{41}N_7O_{19}P_3S$	868.1378	868.1385	-0.81	$C_{15}H_{25}N_2O_6S$	361.1428	361.1428	0.00	16.00	2.29
3-Hydroxy-3-<br>methylbutyryl-CoA	$C_{26}H_{45}N_7O_{18}P_3S$	868.1753	868.1749	0.46	$C_{16}H_{29}N_2O_5S$	361.1792	361.1792	0.00	0.65	0.15
Benzoyl-CoA	$C_{28}H_{41}N_7O_{17}P_3S$	872.1488	872.1487	0.11	$C_{18}H_{25}N_2O_4S$	365.1529	365.1529	0.00	39.63	27.54
3-Oxohexanoyl-CoA	$C_{27}H_{45}N_7O_{18}P_3S$	880.1752	880.1749	0.34	$C_{17}H_{29}N_2O_5S$	373.1793	373.1792	0.27	9.70	0.66
Ethylmalonyl-CoA<br>Methylsuccinyl-CoA<br>Glutaryl-CoA	$C_{26}H_{43}N_7O_{19}P_3S$	882.1533	882.1540	-0.79	$C_{16}H_{27}N_2O_6S$	375.1587	375.1584	0.80	0.22	0.03
3-Hydroxyhexanoyl-CoA	$C_{27}H_{47}N_7O_{18}P_3S$	882.1907	882.1906	0.11	$C_{17}H_{31}N_2O_5S$	375.1949	375.1948	0.27	5.21	
Phenylacetyl-CoA	$C_{29}H_{43}N_7O_{17}P_3S$	886.1639	886.1643	-0.45	$C_{19}H_{27}N_2O_4S$	379.1689	379.1686	0.79	3.76	0.01

Exp.- experimental value, Cal. – calculated (theoretical) value.

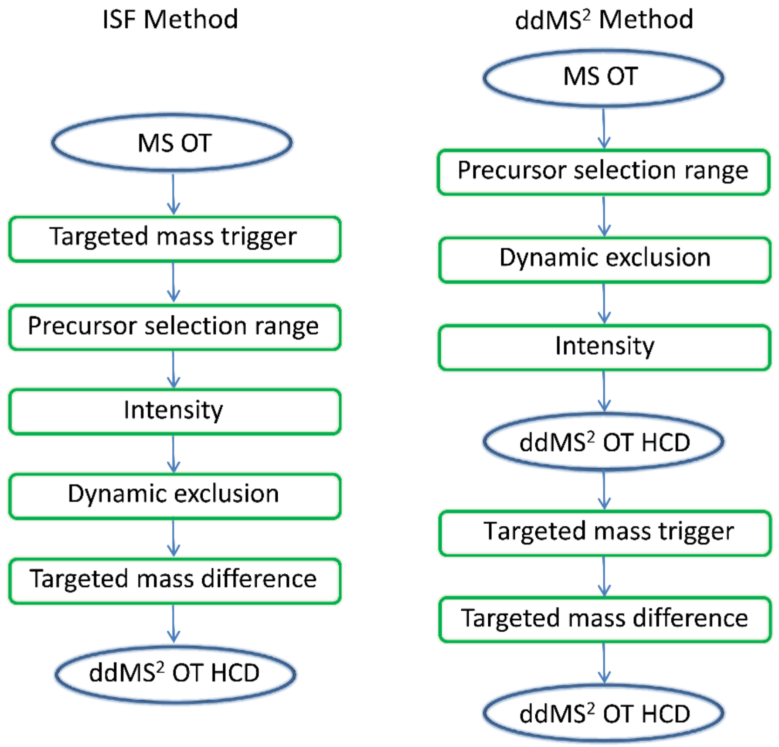

Fig. S1 Representation of the Orbitrap Fusion non-targeted screening methods for the analysis of acyl-CoAs. ISF – in source fragmentation, $ddMS^2$ – data-depending MS/MS, MS – full scan measurement, OT – orbitrap detection, HCD – higher-energy collisional dissociation.

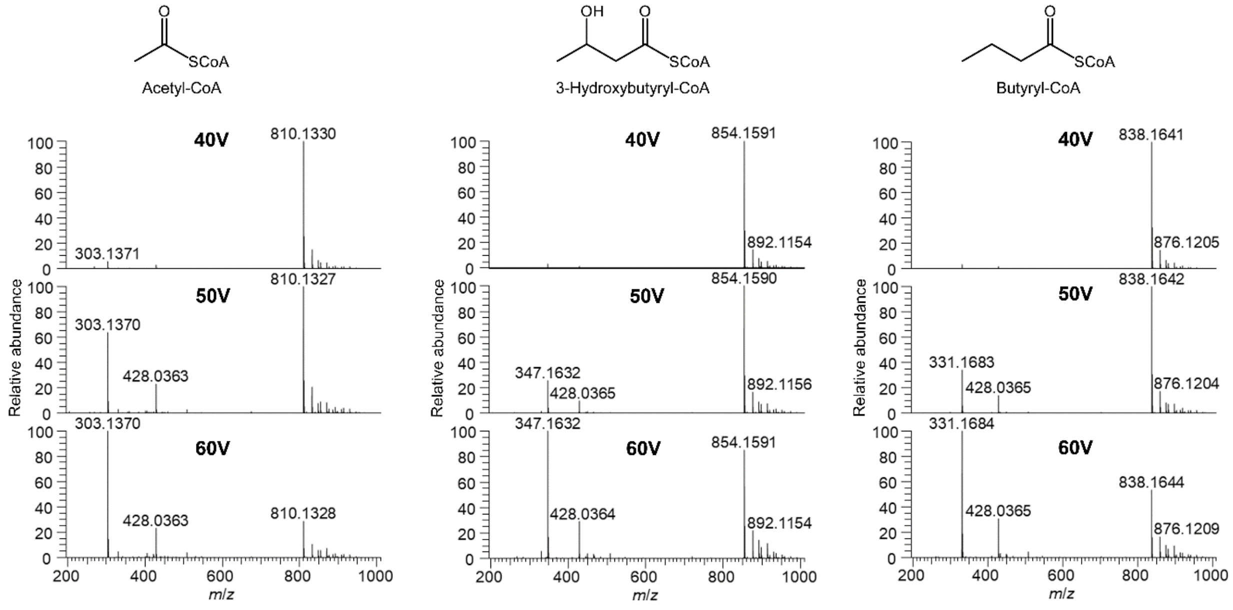

Fig. S2 Mass spectra of acetyl-, 3-hydroxybutyryl- and butyryl-CoA acquired at three different voltage values (bold) applied for in source fragmentation.

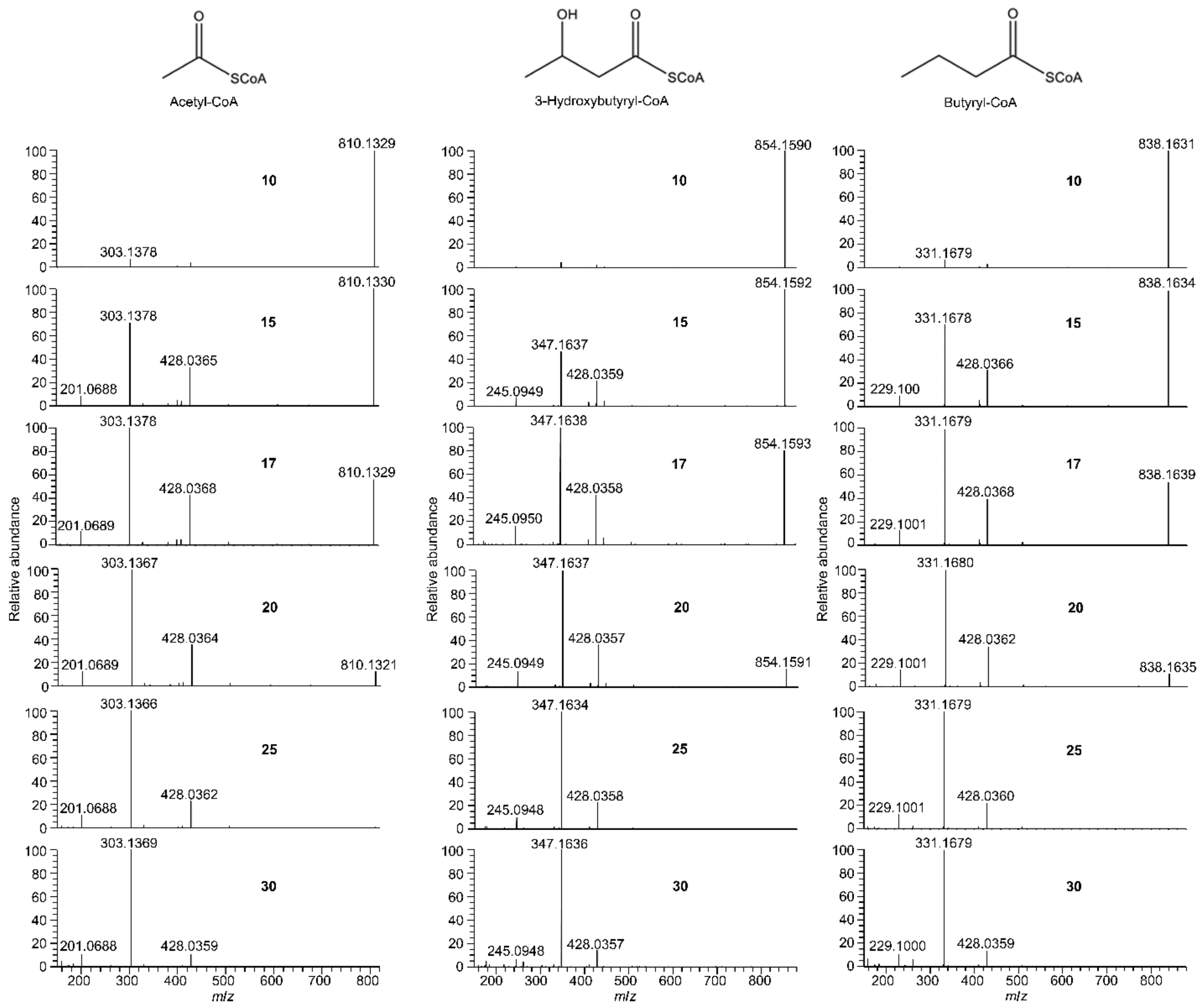

Fig. S3 Mass spectra of acetyl-, 3-hydroxybutyryl- and butyryl-CoA acquired at six different values (bold) applied for HCD cell fragmentation.

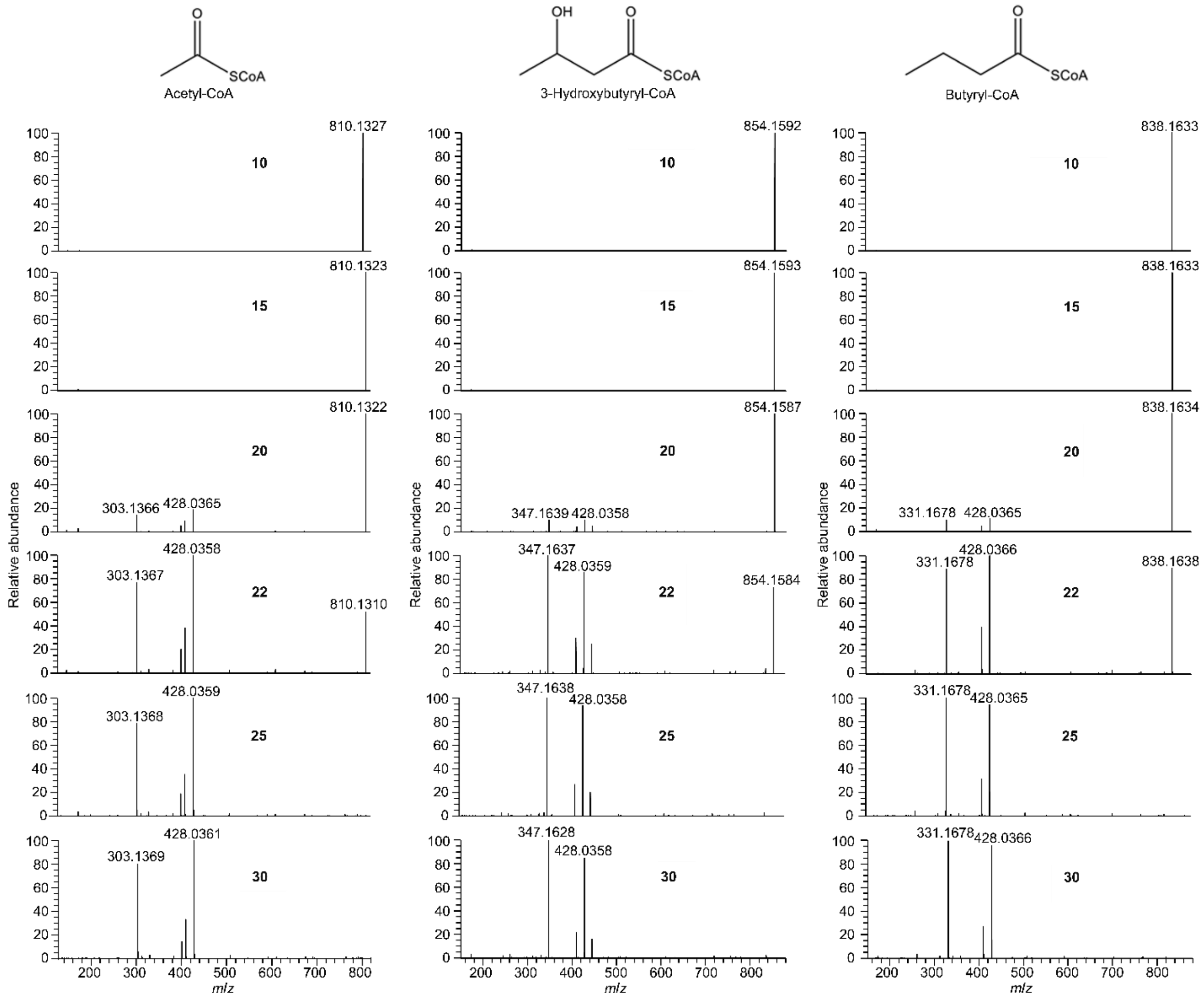

Fig. S4 Mass spectra of acetyl-, 3-hydroxybutyryl- and butyryl-CoA acquired at six different values (bold) applied for CID cell fragmentation.

Table S2 Comparison of acyl-CoA standards analysis using two different screening methods.

Dilution factor:		/		1:2		1:5		1:10		1:50		
Method:		ISF	$ddMS^2$	ISF	$ddMS^2$	ISF	$ddMS^2$	ISF	$ddMS^2$	ISF	$ddMS^2$	
Compound	$[M+H]^+$											Relative intensity[a]
Acetyl-CoA*	810.1331	+	+	+	+	+	+	+	+	-	-	2.38E+06
Propionyl-CoA*	824.1487	+	+	+	+	+	+	+	+	+	+	3.49E+06
Glycolyl-CoA (Hydroxyacetyl-CoA)	826.1280	+	+	+	+	+	+	+	+	-	-	1.77E+06
Crotonyl-CoA*	836.1487	+	+	+	+	+	+	+	+	-	-	3.97E+05
Butyryl-CoA*	838.1644	+	+	+	+	+	+	+	+	+	+	4.41E+06
Isobutyryl-CoA*	838.1644	+	+	+	+	+	+	+	+	+	+	
3-Hydroxypropionyl-CoA	840.1436	+	+	+	+	+	+	+	+	-	-	9,54E+05
Pentenoyl-CoA	850.1644	+	+	+	+	+	+	+	+	-	-	1,95E+06
Acetoacetyl-CoA*	852.1436	+	+	+	+	+	+	+	-	-	-	3,26E+05
2-Methylbutyryl-CoA	852.1796	+	+	+	+	+	+	-	-	-	-	
Isopentanoyl-CoA	852.1796	+	+	+	+	+	+	-	-	-	-	2.15E+05
Pentanoyl-CoA	852.1796	+	+	+	+	+	+	-	-	-	-	
Malonyl-CoA*	854.1231	+	+	+	+	+	-	-	-	-	-	1.05E+05
3-Hydroxybutyryl-CoA*	854.1593	+	+	+	+	+	+	+	+	-	-	1.08E+06
trans-Hexenoyl-CoA	864.1800	+	+	+	+	+	+	-	-	-	-	2.40E+05
Hexanoyl-CoA	866.1957	+	+	+	+	+	+	+	+	+	+	5.32E+06
Succinyl-CoA*	868.1385	+	+	+	+	+	+	+	+	+	+	1.02E+07
Methylmalonyl-CoA*	868.1385	+	+	+	+	+	+	+	+	+	+	
3-Hydroxy-3-methylbutyryl-CoA	868.1748	+	+	+	+	+	+	+	+	+	+	4.59E+06
Benzoyl-CoA	872.1487	+	+	+	+	+	+	+	+	+	-	2.12E+06
2-Methyl-2-hexenoyl-CoA	878.1959	+	+	+	+	-	+	-	-	-	-	4.88E+05
Glutaconyl-CoA*	880.1385	+	+	+	+	+	+	+	-	-	-	2.00E+05
3-Oxohexanoyl-CoA	880.1752	+	+	+	+	+	+	+	+	+	-	1.70E+06
Ethylmalonyl-CoA	882.1542	+	+	+	+	+	+	+	+	-	-	
Glutaryl-CoA	882.1544	+	+	+	+	+	+	+	+	-	-	1.82E+06
Methylsuccinyl-CoA	882.1544	+	+	+	+	+	+	+	+	-	-	
3-Hydroxyhexanoyl-CoA	882.1906	+	+	+	+	+	+	+	+	-	-	1.38E+06
Phenylacetyl-CoA	886.1639	+	+	+	+	+	+	-	-	-	-	2.43E+05
2-Methyl-3-oxohexanoyl-CoA	894.1907	+	+	+	+	+	+	+	-	-	-	3.33E+05
3-Hydroxy-2-methylhexanoyl-CoA	896.2057	+	+	+	+	-	-	-	-	-	-	7.69E+04
4-Methyl-3-oxooctanoyl-CoA	922.2224	+	+	+	+	-	-	-	-	-	-	1.66E+05
3-Hydroxy-4-methyloctanoyl-CoA	924.2380	+	+	+	+	+	+	-	-	-	-	6.98E+05

[a]-value for the extracted $[M+H]^+$ ion in undiluted sample, *- commercial standards.
The differences in results among the methods are highlighted in grey.

Table S3 Acyl-CoAs identified in the extracts of the strain HxN1, analyzed with two different screening methods.

Amount of sample injection:		10 µL				5 µL				2 µL			
Method:		ISF		ddM[2]		ISF		ddM[2]		ISF		ddM[2]	
Sample:		½OD_{max}	OD_{max}	½OD_{max}	OD_{max}	½OD_{max}	OD_{max}	½OD_{max}	OD_{max}	½OD_{max}	OD_{max}	½OD_{max}	OD_{max}
Compounds	$[M+H]^+$												
Acetyl-CoA[a]	810.1329	+	+	+	+	+	+	+	+	+	+	+	+
Propionyl-CoA[a]	824.1487	+	+	+	+	+	+	+	+	+	+	+	+
Crotonyl-CoA[a]	836.1484	+	+	+	-	+	+	+	-	+	+	+	-
Isobutyryl-CoA[a]	838.1637	+	+	+	+	+	+	+	+	+	+	+	+
Butyryl-CoA[a]	838.1637	+	+	+	+	+	+	+	+	+	+	+	+
3-Hydroxypropionyl-CoA[b]	840.1439	+	+	+	+	+	+	-	+	-	-	-	+
Acetoacetyl-CoA[a]	852.1437	+	+	+	+	+	+	-	+	-	-	-	-
2-Methylbutyryl-CoA[b]	852.1796	+	-	+	-	+	-	+	-	+	-	+	-
Isopentanoyl-CoA[b]	852.1796	+	-	+	-	+	-	+	-	+	-	+	-
Pentanoyl-CoA[b]	852.1796	+	+	+	+	+	+	+	+	+	+	+	+
Malonyl-CoA[a]	854.1231	+	+	+	+	+	+	+	+	+	+	+	+
3-Hydroxybutyryl-CoA[a]	854.1592	+	+	+	+	+	+	+	+	+	+	+	+
trans-2-Hexenoyl-CoA[b]	864.1800	+	+	+	+	+	+	+	-	+	+	+	-
Hexanoyl-CoA[b]	866.1962	+	+	+	+	+	+	+	+	+	+	+	+
Succinyl-CoA[a]	868.1378	+	+	+	+	+	+	+	+	+	+	+	+
Methylmalonyl-CoA[a]	868.1378	+	+	+	+	+	+	+	+	+	+	+	+
3-Methyl-3-hydroxybutyryl-CoA[b]	868.1753	+	+	+	+	+	+	+	+	+	+	+	+
Benzoyl-CoA[b]	872.1488	+	+	+	+	+	+	+	+	+	+	+	+
3-Oxohexanoyl-CoA[b]	880.1752	+	+	+	-	-	+	-	-	-	-	-	-
Ethylmalonyl-CoA[b]	882.1533	+	+	+	+	+	+	+	+	-	+	-	+
Methylsuccinyl-CoA[b]	882.1533	+	-	+	-	+	-	+	-	-	-	-	-
Glutaryl-CoA[a]	882.1533	+	+	+	+	+	+	+	+	-	+	-	+
3-Hydroxyhexanoyl-CoA[b]	882.1907	+	-	+	-	+	-	+	-	+	-	+	-
Phenylacetyl-CoA[b]	886.1639	+	+	+	+	+	+	+	+	+	+	+	+

The differences in results among the methods are highlighted in grey.

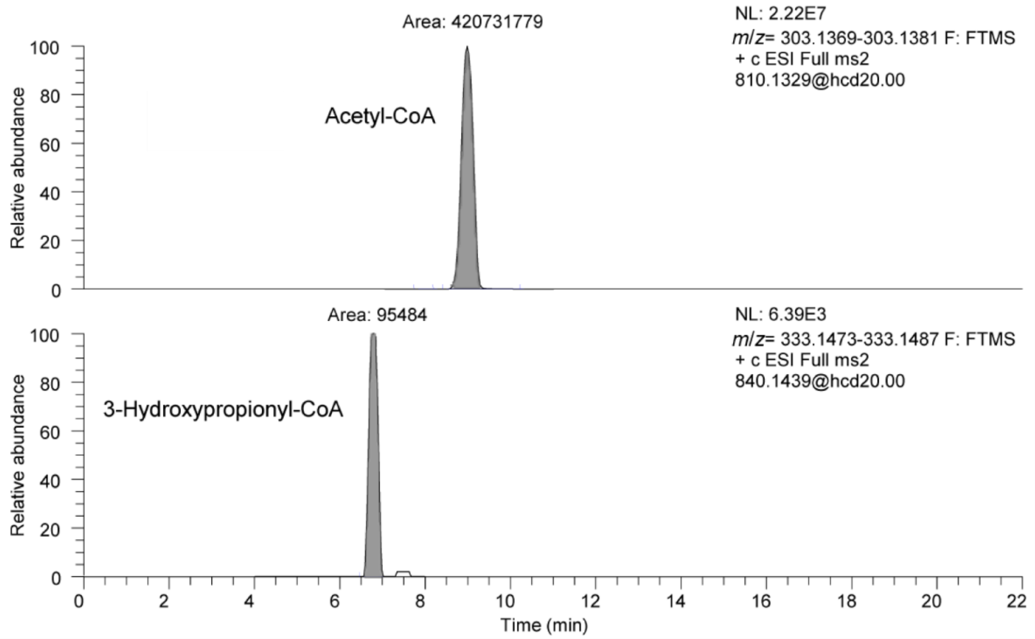

Fig. S5 Extracted ion chromatograms of acetyl-CoA and 3-hydroxypropionyl-CoA detected in the targeted screening of the extract of cells of strain HxN1 grown with hexanoate and harvested at OD_{max}. The integrated peak areas show the ratio of 2.3 x 10^{-4}.

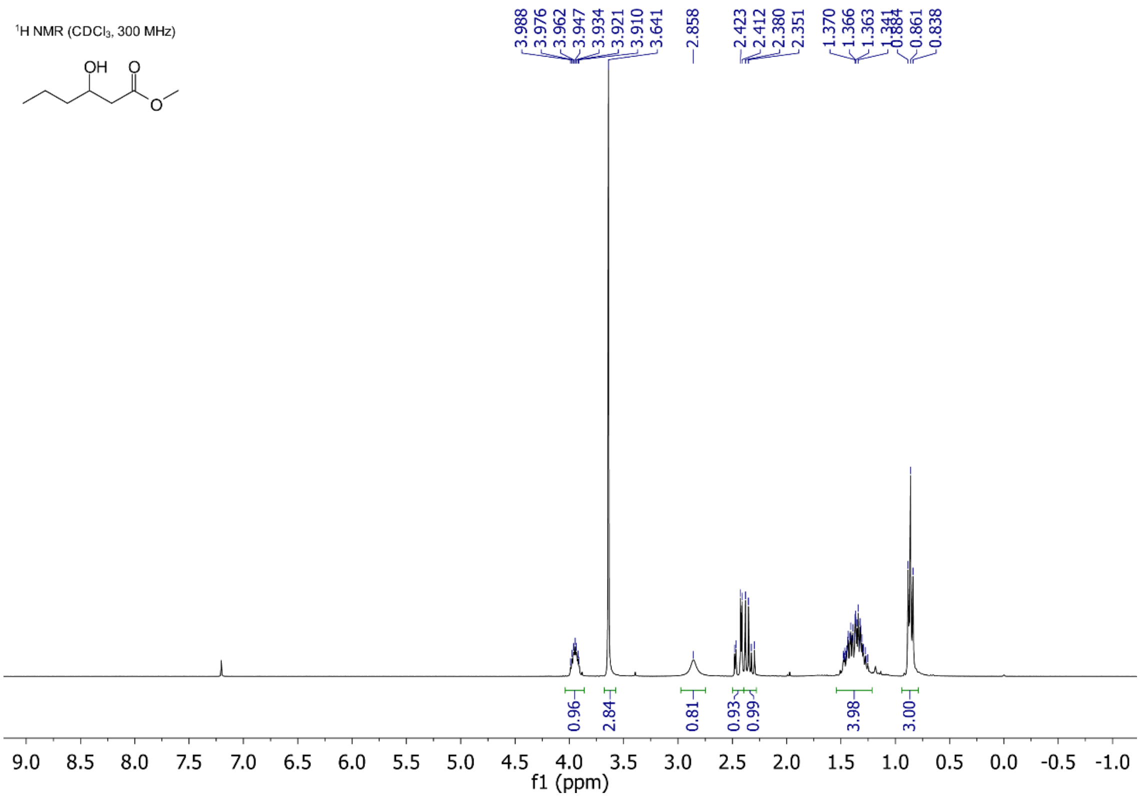

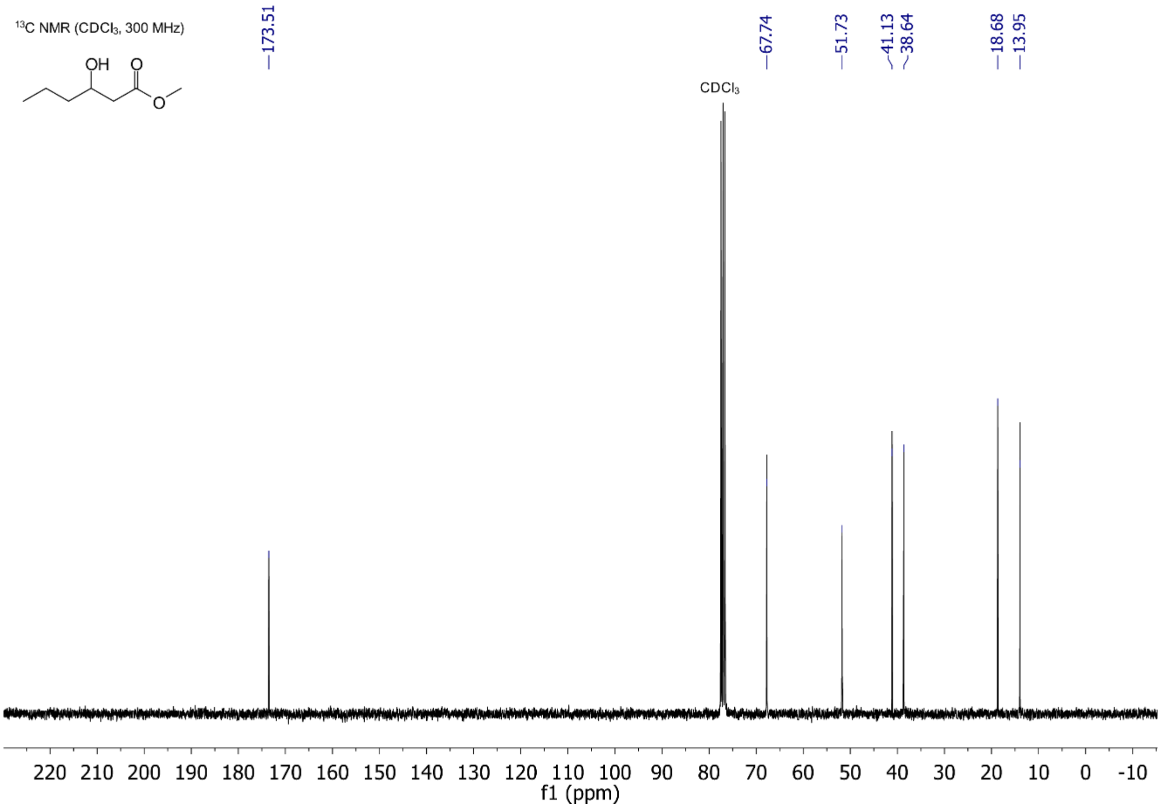

Fig. S6 ^{1}H (above) and ^{13}C (below) NMR spectra of methyl 3-hydroxyhexanoate.

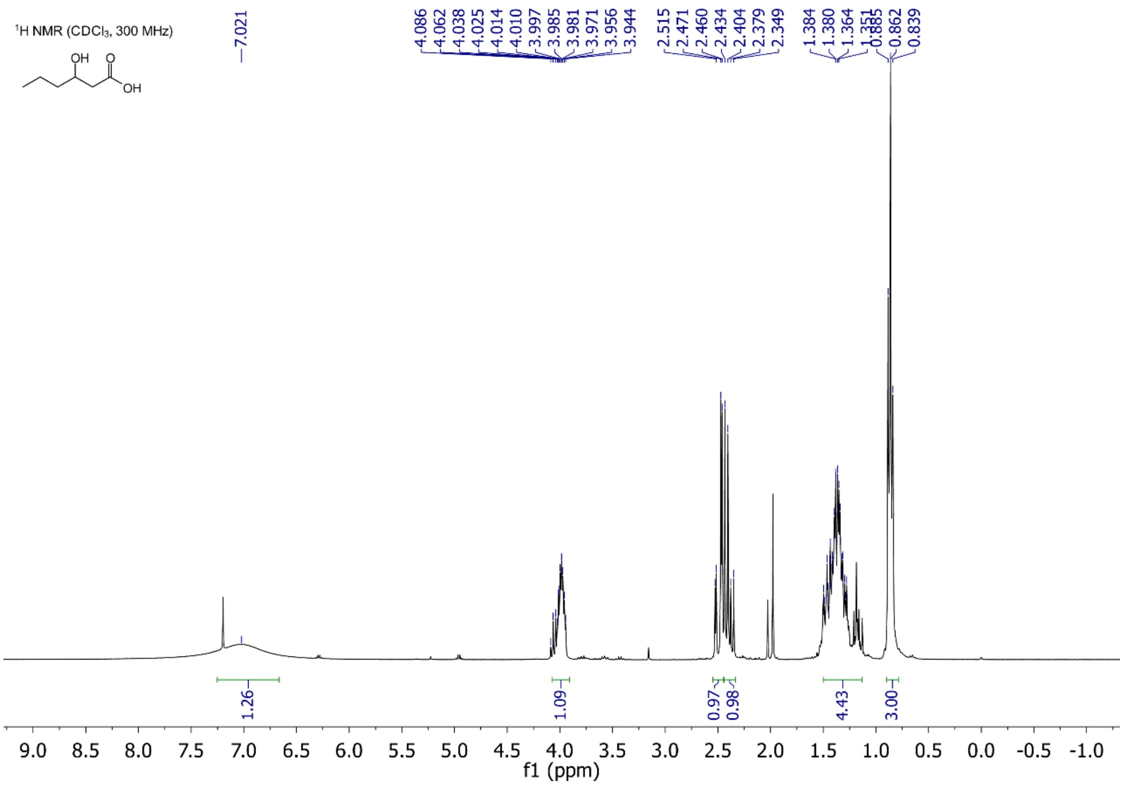

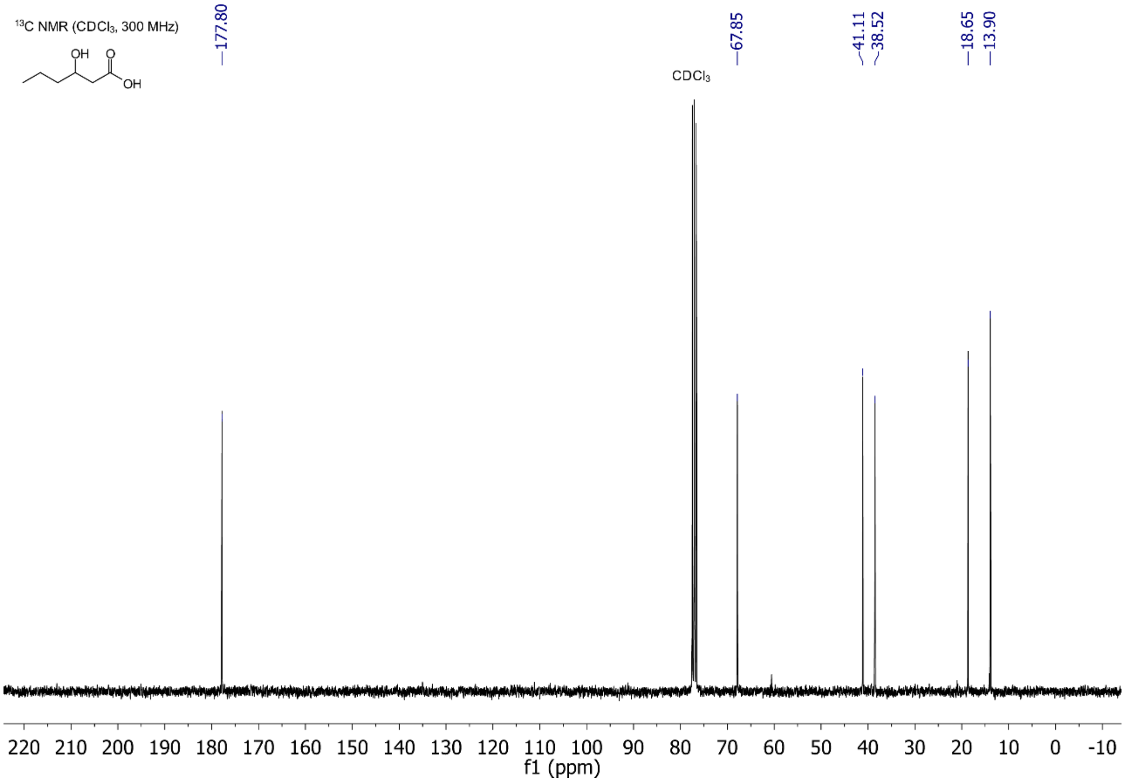

Fig. S7 ^{1}H (above) and ^{13}C (below) NMR spectra of 3-hydroxyhexanoic acid.

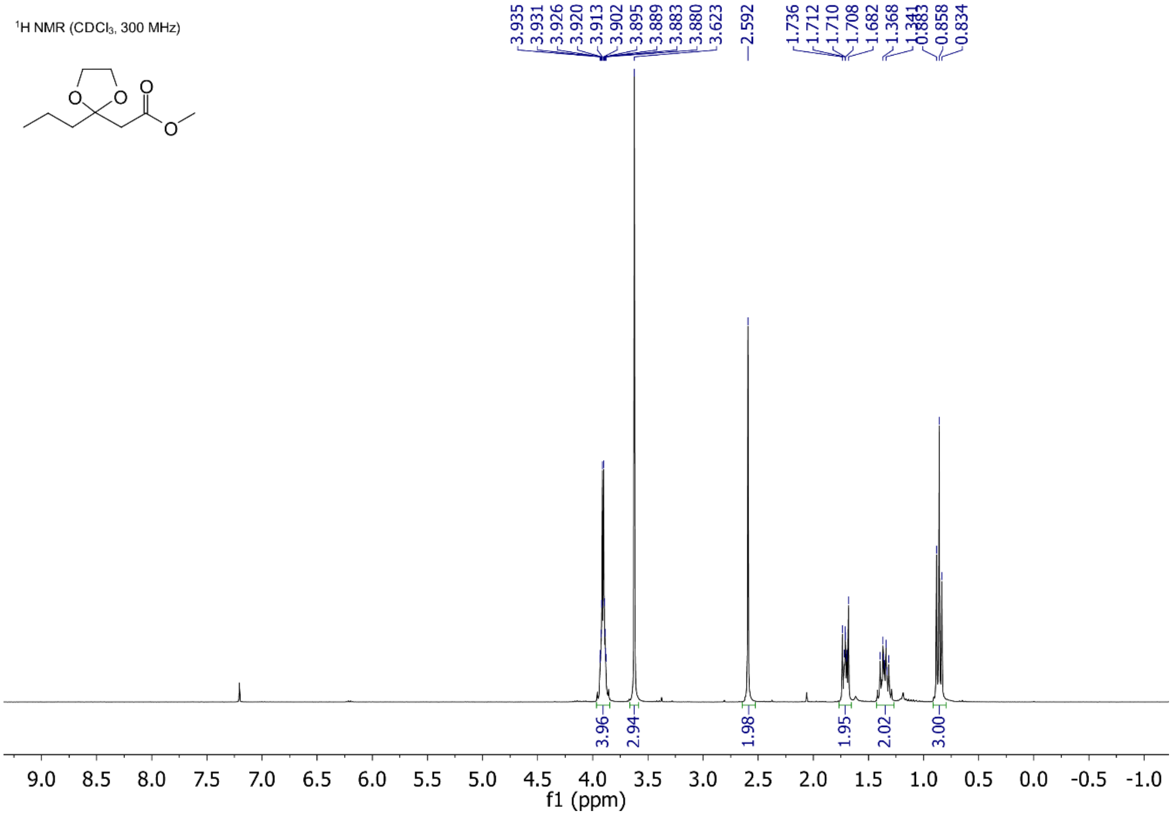

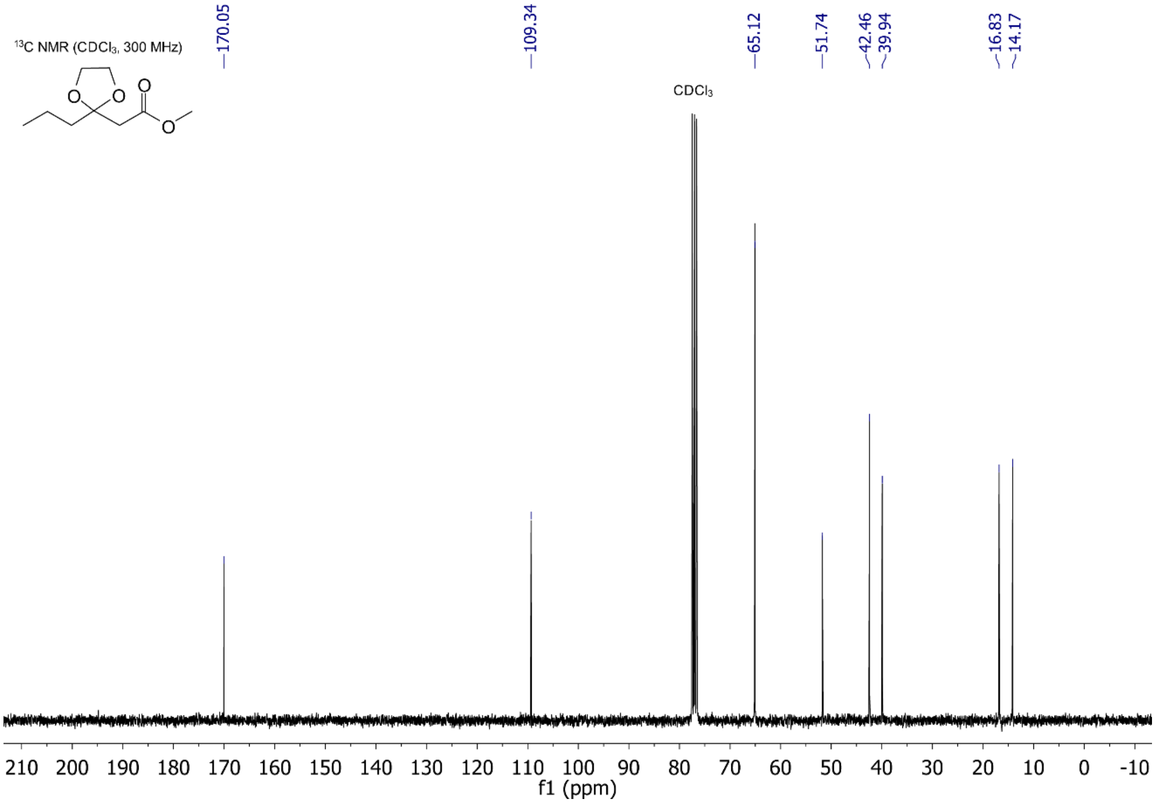

Fig. S8 ^{1}H (above) and ^{13}C (below) NMR spectra of methyl 2-(2-propyl-1,3-dioxolan-2-yl)acetate.

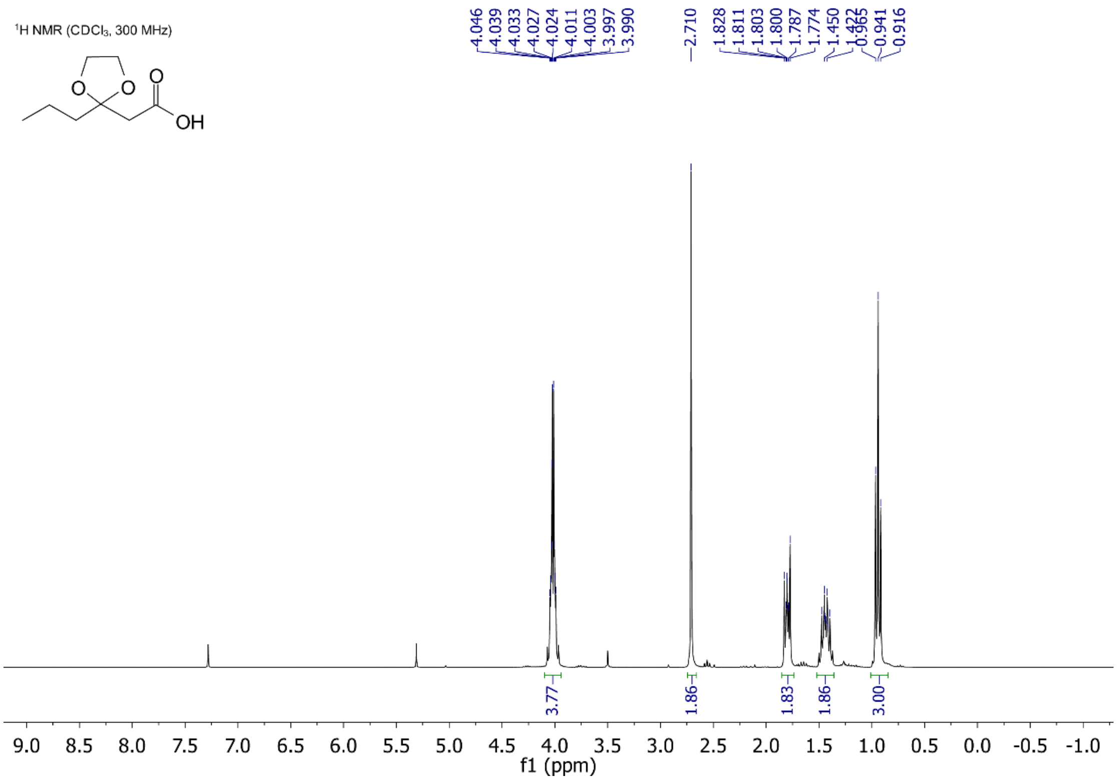

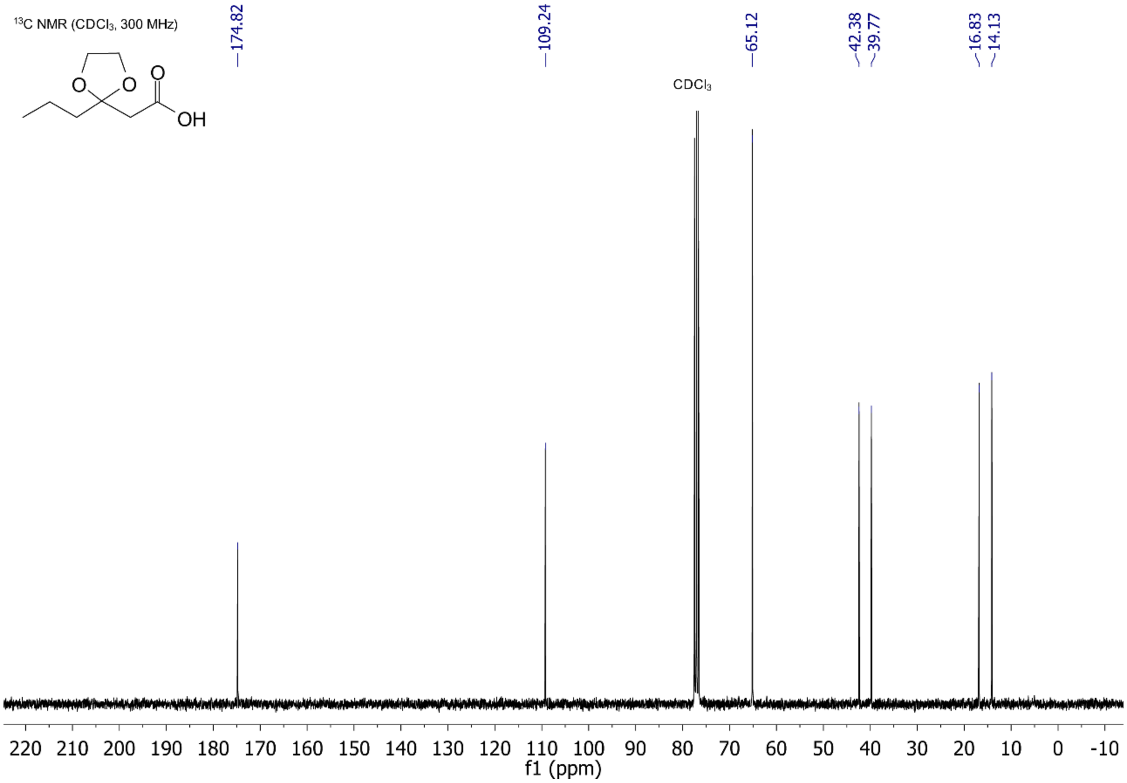

Fig. S9 ^{1}H (above) and ^{13}C (below) NMR spectra of 2-(2-propyl-1,3-dioxolan-2-yl)acetic acid.

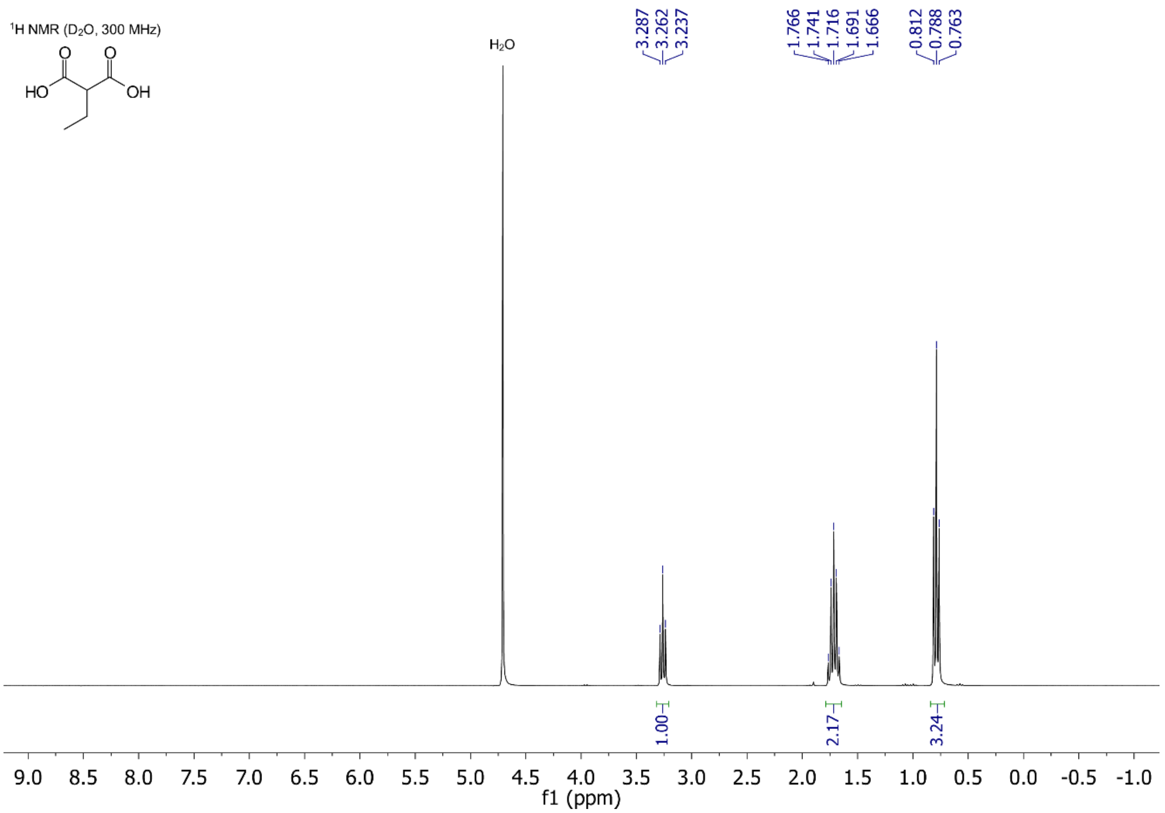

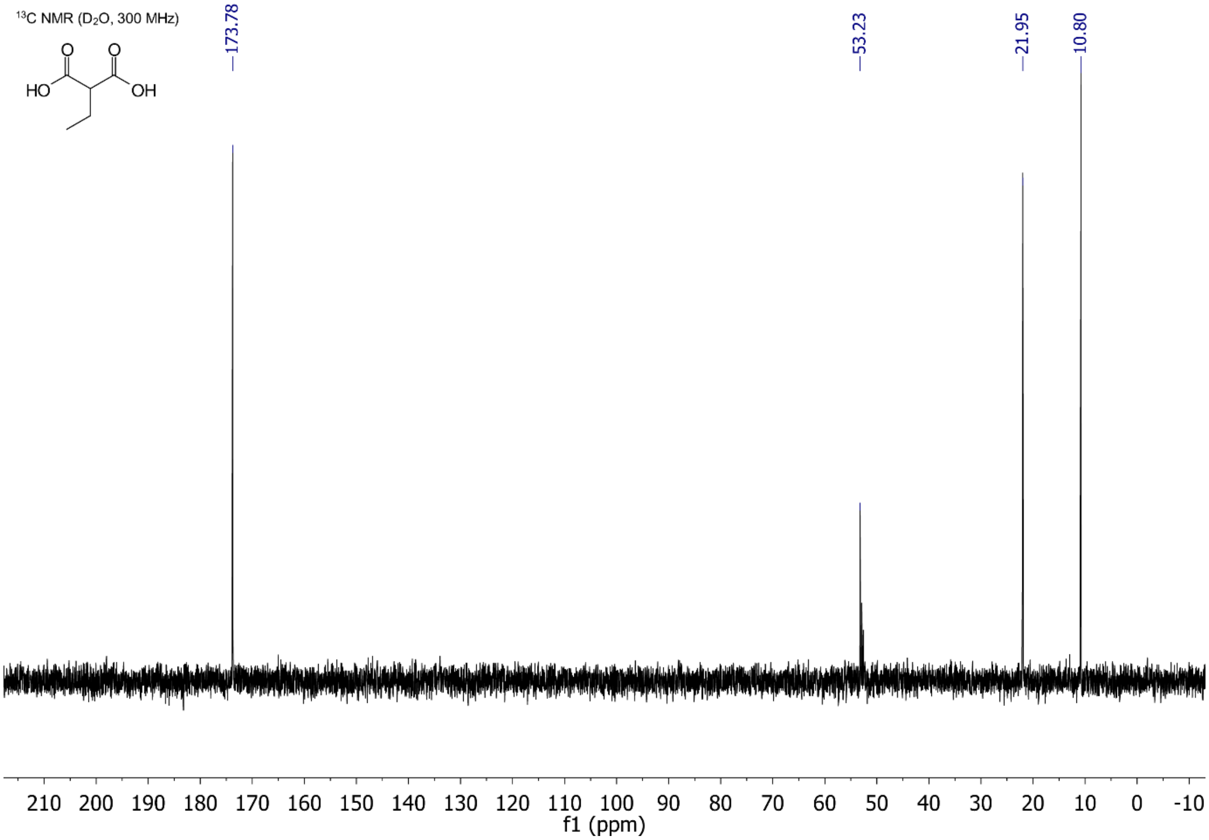

Fig. S10 ^{1}H (above) and ^{13}C (below) NMR spectra of ethyl malonic acid.

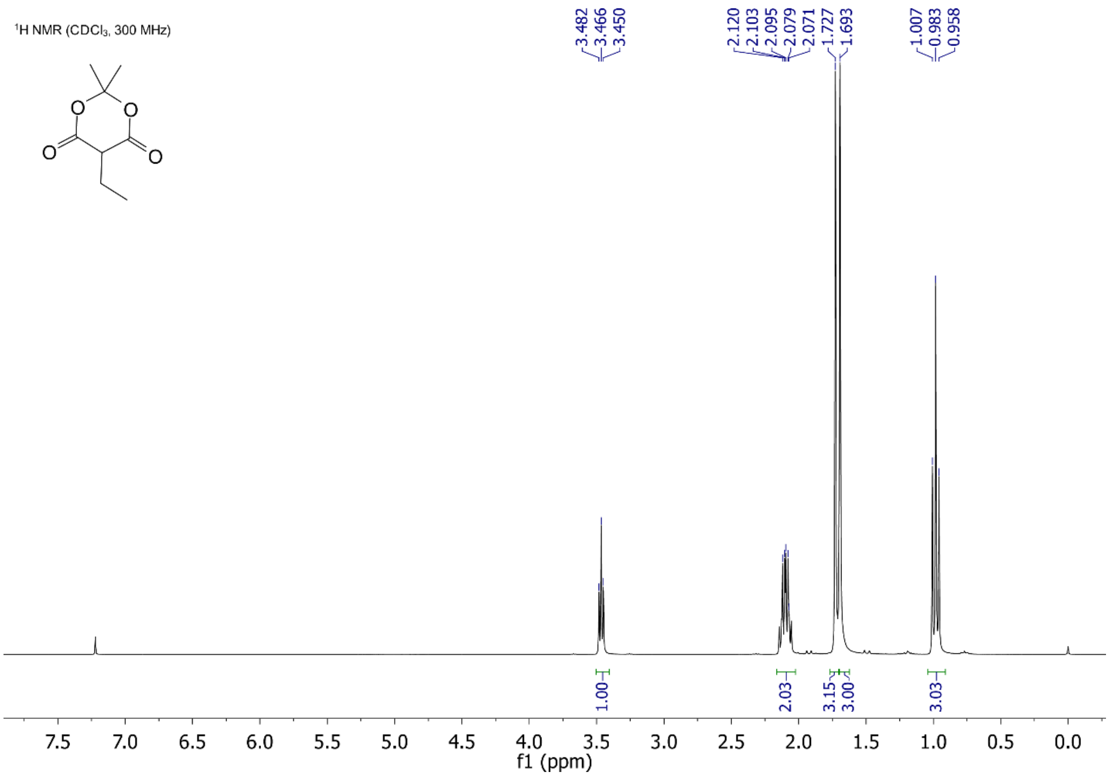

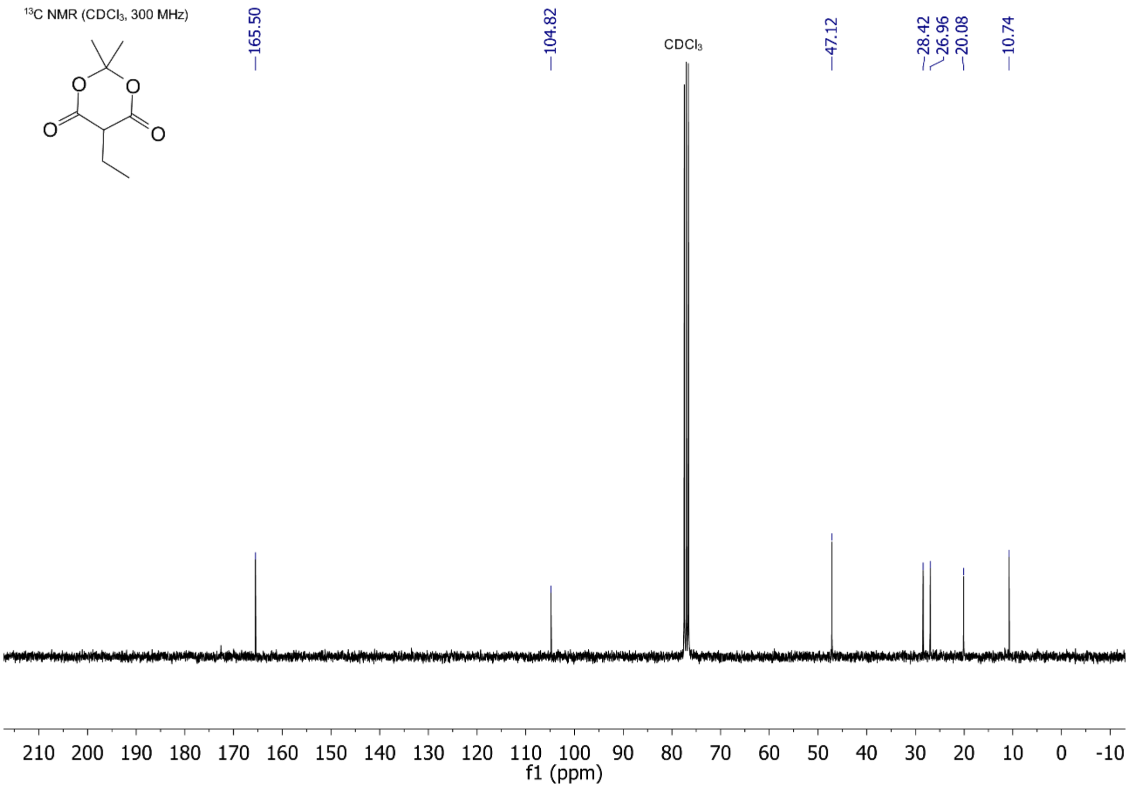

Fig. S11 ^{1}H (above) and ^{13}C (below) NMR spectra of 2,2-dimethyl-5-ethyl-1,3-dioxan-4,6-dion.

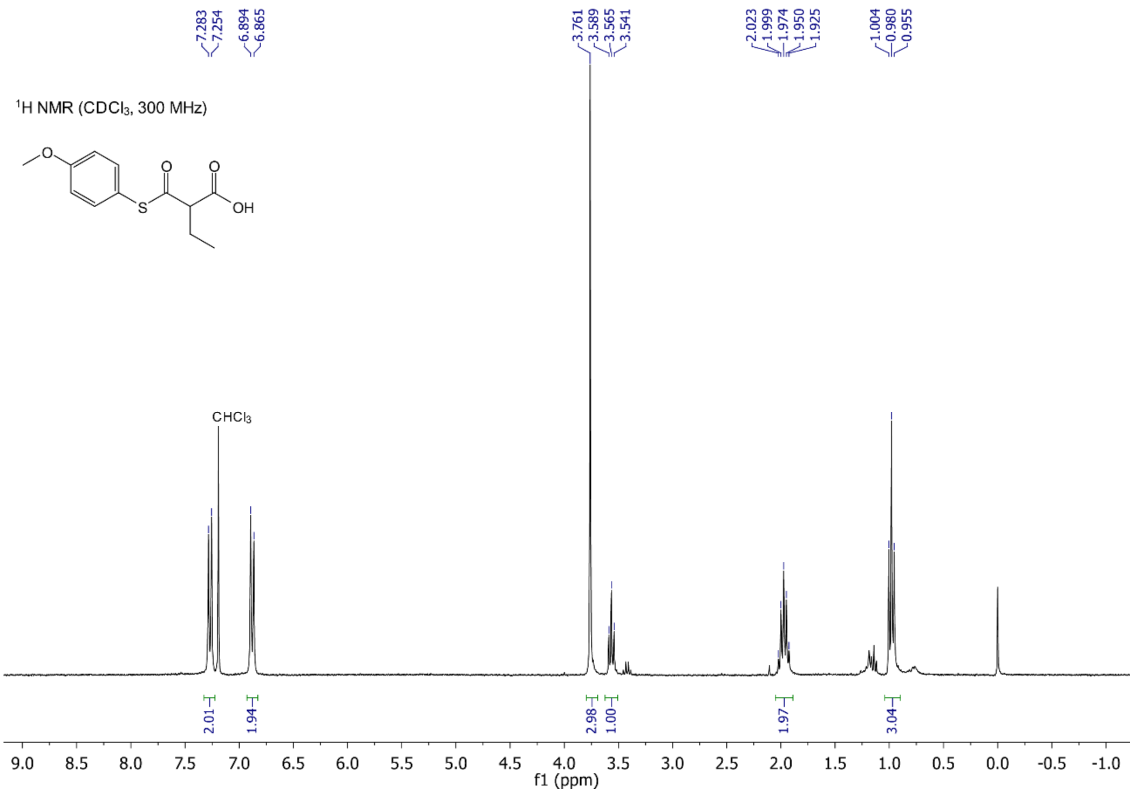

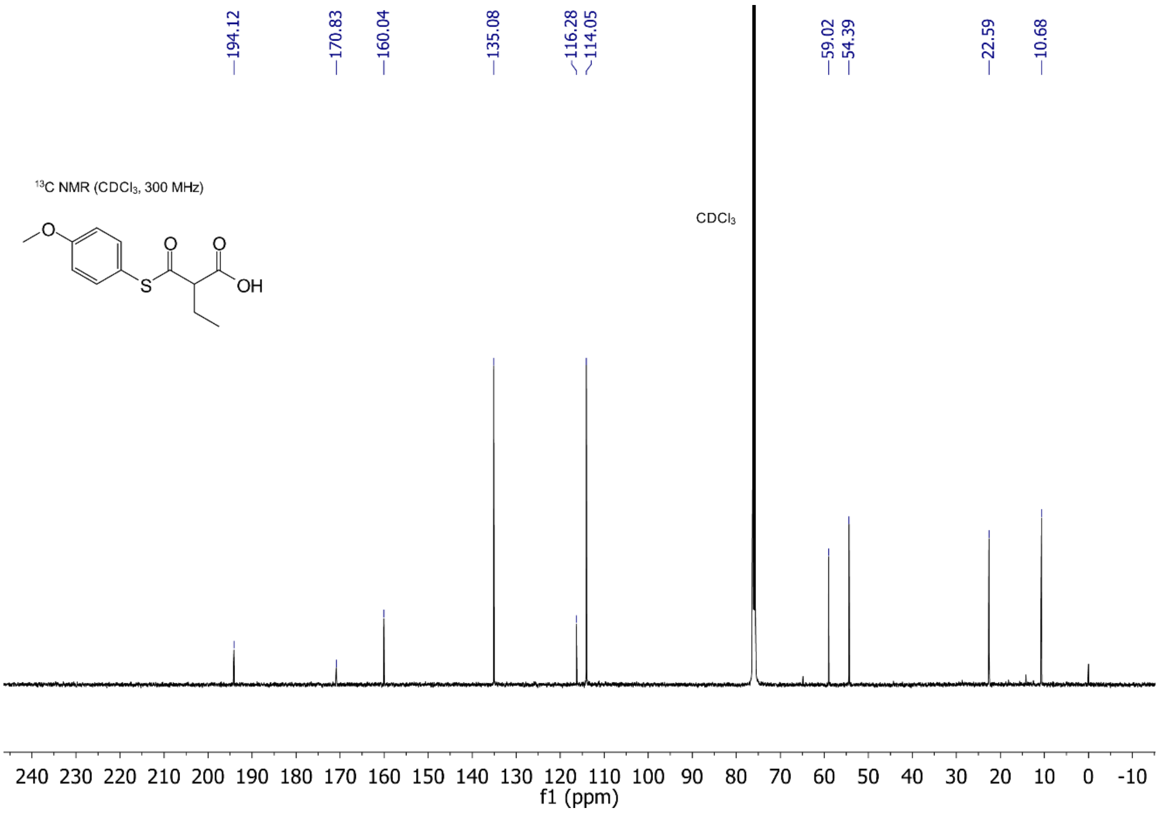

Fig. S12 ^{1}H (above) and ^{13}C (below) NMR spectra of 2-((4-methoxyphenylthio)carbonyl)butanoic acid.

References

1. Kawaguchi, A.; Yoshimura, T.; Okuda, S. A New Method for the Preparation of Acyl-CoA. *J. Biochem.* 1981; 89: 337–339.

2. Peter, D. M.; Vögeli, B.; Cortina, N. S.; Erb, T. J. A Chemo-Enzymatic Road Map to the Synthesis of CoA Esters. *Molecules* 2016; 21 (4): 1–11.

3. Kanchanabanca, C.; Tao, W.; Hong, H.; Liu, Y.; Hahn, F.; Samborskyy, M.; Deng, Z.; Sun, Y.; Leadlay, P. F. Unusual Acetylation-Elimination in the Formation of Tetronate Antibiotics. *Angew. Chemie - Int. Ed.* 2013; 52 (22): 5785–5788.

4. Engl, O. D.; Saadi, J.; Cosimi, E.; Wennemers, H. Synthesis of Monothiomalonates – Versatile Thioester Enolate Equivalents for C–C Bond Formations. *Helv. Chim. Acta* 2017; 100 (11): 1–14.
